# Transcriptome Analysis Reveals Molecular Mechanisms under Salt Stress in Leaves of Foxtail Millet (*Setaria italica* L.)

**DOI:** 10.3390/plants11141864

**Published:** 2022-07-18

**Authors:** Fei Han, Mingjie Sun, Wei He, Shuqing Guo, Jingyi Feng, Hui Wang, Quangang Yang, Hong Pan, Yanhong Lou, Yuping Zhuge

**Affiliations:** 1 National Engineering Research Center for the Efficient Utilization of Soil and Fertilizer, College of Resources and Environment, Shandong Agricultural University, Tai’an 271018, China; hanfeiyanzhou@163.com (F.H.); mingjiesun1995@163.com (M.S.); hewei0534@163.com (W.H.); sdaufjy2018@163.com (J.F.); huiwang@sdau.edu.cn (H.W.); sttzzy@sdau.edu.cn (Q.Y.); panhong6239@163.com (H.P.); 2 College of Agronomy, Northwest A&F University, Xianyang 712100, China; gsq055069@nwafu.edu.cn

**Keywords:** foxtail millet, salt stress, salt tolerance identification, transcriptome, DEGs, RNA

## Abstract

Foxtail millet (*Setaria italica* L.) is an important cereal for managing future water scarcity and ensuring food security, due to its strong drought and salt stress resistance owing to its developed root system. However, the molecular responses of foxtail millet leaves to salt stress are largely unknown. In this study, seeds of 104 foxtail millet accessions were subjected to 0.17 mol·L^−1^ NaCl stress during germination, and various germination-related parameters were analyzed to derive 5 salt-sensitive accessions and 13 salt-tolerant accessions. Hong Gu 2000 and Pu Huang Yu were the most salt-tolerant and salt-sensitive accessions, respectively. To determine the mechanism of the salt stress response, transcriptomic differences between the control and salt-treated groups were investigated. We obtained 2019 and 736 differentially expressed genes under salt stress in the salt-sensitive and salt-tolerant accessions, respectively. The transcription factor families bHLH, WRKY, AP2/ERF, and MYB-MYC were found to play critical roles in foxtail millet’s response to salt stress. Additionally, the down-regulation of ribosomal protein-related genes causes stunted growth in the salt-sensitive accessions. The salt-tolerant accession alleviates salt stress by increasing energy production. Our findings provide novel insights into the molecular mechanism of foxtail millet’s response to salt stress.

## 1. Introduction

Soil salinization poses significant ecological and environmental challenges in dry farming areas [1,2]. According to the World Food and Agriculture Organization and the Scientific and Educational Organization, salinized land covers approximately 950 million ha, accounting for 10% of the global land area [3,4]. Although salinized land has great agriculture potential due to its flat terrain and deep soil layer suitable for farming, the high salt content, high alkalinity, poor physical structure, and lack of nutrients in saline–alkali soil hinder the normal growth of plants and seriously threaten agricultural production [5]. Specifically, the lack of suitable salt-tolerant crops and limited understanding of the salt-tolerant mechanism restricts the development and utilization of salinized land [6,7].

Foxtail millet (*Setaria italica* L.; family Gramineae) is one of the oldest crops grown in the world, dating back some 7400–7935 years in northern China [8]. Foxtail millet occurs naturally in arid areas and semi-arid regions, with Asia and Africa producing nearly 95% of global production [9,10]. Foxtail millet contains a wide range of vitamins, amino acids, fats, minerals, and crude fiber [11,12]. In addition to its nutritional properties, foxtail millet has also been shown to provide several health benefits, such as the prevention of cancer and cardiac–cerebral vascular disease [13]. Therefore, the cultivation and processing of foxtail millet into high-value-added food can contribute significantly to the economic development of many countries in Asia and Africa [10]. More importantly, foxtail millet has a suitable adaptive ability to change environmental conditions, especially under increasingly intensified land aridification. Foxtail millet has a great potential for salt tolerance due to its strong fibrous root system, high water utilization rate, strong drought tolerance, and salt tolerance potential [14,15]. Nahar et al. [16] found that the salt tolerance of a foxtail millet accession could maintain higher dry matter and grain yield at 6 dS·m^−1^ salt level. Krishnamurthy et al. [17] identified 155 foxtail millet accessions in India on soil saturated with 100 mM NaCl, of which 13 accessions were highly tolerant. However, the salt-tolerance mechanism is unclear and the lack of genetic information related to salt tolerance restricts the popularization of foxtail millet cultivation in salinized land.

Transcriptome analysis is an important method for revealing the molecular mechanism underlying plant stress resistance [18]. Transcriptome refers to the collection of transcriptional products of a specific tissue or cell in a certain state [19,20]. Comparing differentially expressed genes (DEGs) of accessions with different salt tolerances by transcriptome technology can reveal the changes in plant expression pathways and the salt-tolerance mechanism. 

The publication of the foxtail millet genome provided an opportunity to better understand the salt tolerance mechanism. Suppression subtractive hybridization of the whole seedlings allows the detection of effective signal-perception mechanisms in tolerant cultivars for metabolic adjustments under high saline conditions, and 81 differentially expressed transcripts not reported in other plants were identified [21]. Based on the differences in root transcription between the two foxtail millets studied by Pan et al. [22], the salt tolerance of foxtail millet was attributed to highly efficient ion channels and excellent antioxidant capacity. Nevertheless, foxtail millet leaves are more sensitive to salt stress at the seedling stage.

In this study, salt tolerance during germination and seeding of foxtail millet accessions in China was analyzed and evaluated to determine the salt-tolerant and salt-sensitive accessions. Then, the Illumina technology platform was employed to evaluate the gene expression changes in foxtail millet leaves during salt stress treatment at the molecular level. Through functional annotation of the transcriptome, DEGs in foxtail millet under salt stress were identified. The present study also reports the effects of salt stress on the expression of hub genes in foxtail millet leaf cells, which could provide both a theoretical and experimental basis for further work.

## 2. Results

### 2.1. Salt Tolerance Identification

The RGR, RGP, RGI, RPL, and RRL of 104 foxtail millet accessions (Table S1) at the germination stage were reduced under 0.17 mol·L^−1^ NaCl, suggesting that the foxtail millet underwent salt stress during germination. Based on fuzzy mathematics analysis for RGR, RGP, RGI, RPL, and RRL of 104 foxtail millet accessions during the germination stage, 5 salt-tolerant accessions and 13 salt-sensitive accessions were selected.

To determine the most salt-tolerant and salt-sensitive accessions, 5 salt-tolerant and 13 salt-sensitive accessions from the initial screening were subjected to secondary salt-tolerance identification. The 18 accessions were cultured to the seedling stage; the changes in relative biomass, relative growth rate, relative chlorophyll content, and relative K^+^/Na^+^ ratio under 0.13, 0.17, and 0.22 mol·L^−1^ NaCl concentrations were evaluated (Table 1 and Table S2). According to fuzzy mathematics analysis, FM6 ranked third, second, and first at 0.13, 0.17, and 0.22 mol·L^−1^ NaCl, respectively, which was the most salt-tolerant accession; FM90 was the most salt-sensitive accession, with the worst performance under all NaCl concentrations. 

### 2.2. Transcriptome Assembly and Expression Analysis of DEGs

Between the salt-sensitive accession with salt stress (SS) library and salt-sensitive accession without salt stress (SC) library, the screened DEGs were labeled as SS/SC, while the screened DEGs between the salt-tolerant accession with salt stress (TS) library and salt-tolerant accession without salt stress (TC) library were labeled as TS/TC. Compared with SC, a total of 1176 genes were down-regulated in SS, 843 genes were up-regulated, and the expression of 21,741 genes was not significantly enriched. Compared with TC, 345 genes were down-regulated in SC treatment, 391 were up-regulated, and the expression of 21,004 did not differ significantly. There were 295 common DEGs in the SS/SC and TS/TC.

### 2.3. Gene Ontology (GO) Classification of DEGs

**Figure 1:**
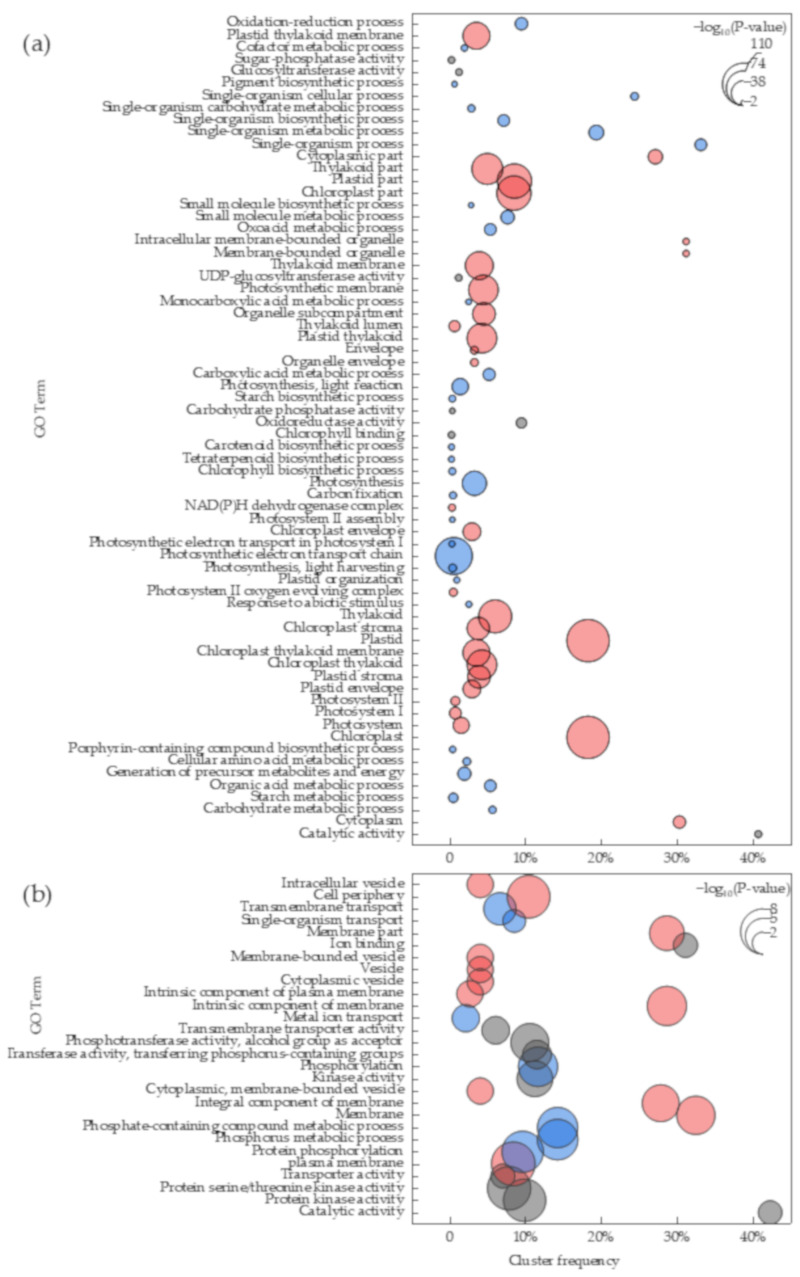
Enrichment analysis of GO between SS/SC (**a**) and TS/TC (**b**). The red, blue, and gray colors represent cellular component, biological process, and molecular function, respectively. Cluster frequency is the proportion of DEGs annotated to a term to the total DEGs annotated to GO database.

For the most salt-sensitive accession, 68 GO terms were significantly enriched (*p* < 0.01), of which 32, 29, and 7 terms were related to biological process, cellular component, and molecular function, respectively (Figure 1). The terms with the most significant change were related to the photoreaction stage in the cellular component, namely, the chloroplasts in the plastid and the photosynthetic and thylakoid membranes in the thylakoid. GO terms enriched in the biological process were related to photosynthesis and single-organism processes. The change in molecular function terms of the salt-sensitive accession was largely in catalytic activity.

For the most salt-tolerant accession, 28 terms were significantly enriched (*p* < 0.01), of which 7, 12, and 9 terms were related to biological process, cellular component, and molecular function, respectively (Figure 1). Changes in the biological process terms of the salt-tolerant accession were primarily in phosphorus metabolics and transmembrane transports (especially metal ions), which affect ion homeostasis. For cellular components, most of the changes in the GO terms were related to the intrinsic components (especially the integral component) of the cell membrane and plasma membrane. In addition, five terms associated with vesicles were significantly enriched, indicating that the salt-tolerant accession also changed the ion permeability. For molecular function terms, nine GO terms were enriched, mainly for “ion binding”, “protein kinase activity”, and “transporter activity”.

### 2.4. Functional Annotation of DEGs Using the Kyoto Encyclopedia of Genes and Genomes (KEGG) Database

**Figure 2:**
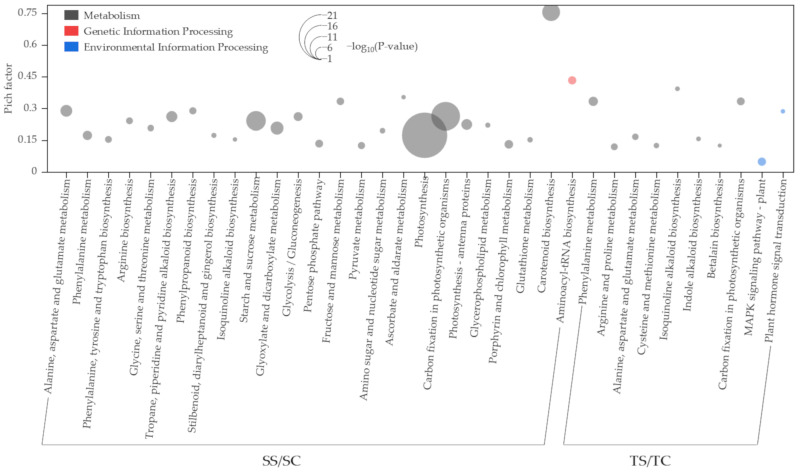
Enrichment analysis of KEGG.

To better understand the biological functions of DEGs, the Gene Ontology database of foxtail millet was used to enrich and analyze genomic transcriptional differences and annotate them. In SS/SC, DEGs of 111 altered pathways were annotated, among which 25 pathways (154 DEGs were down-regulated and 90 DEGs were up-regulated) were significantly enriched (*p* ≤ 0.01) (Figure 2, Table S3). Twenty-four significantly enriched pathways belonged to metabolism, and one pathway was annotated for genetic information processing. Similar to the GO results, the pathways with the most numbers and the highest change degree were related to photosynthesis.

In TS/TC processing, 105 altered pathways were annotated, among which 10 pathways (15 DEGs were down-regulated and 25 DEGs were up-regulated) were significantly enriched (*p* ≤ 0.01) (Figure 2, Table S3). For the enrichment pathway, except for two signal conversions related to environmental information processing, all pathways were related to metabolism. The highest degree of enrichment was related to amino acid metabolism, signal transduction, and photosynthesis.

Four KEGG pathways (sita00710, sita00250, sita00360, and sita00950) were significantly enriched in both salt-tolerant and salt-sensitive accessions (*p* ≤ 0.01) (Figure 2, Table S3). The degree of change in the salt-tolerant accession was higher than in the salt-sensitive accession in most KEGG pathways, with common enrichment pathways in both types of accessions. More importantly, “photosynthesis (sita00195)”, which was the greatest change pathway in the salt-sensitive accession, was not significantly changed in the salt-tolerant accession; “photosynthesis–antenna proteins”, which significantly changed in the salt-sensitive accession, were not detected in the salt-tolerant accession.

### 2.5. DEGs Related to Photosynthesis 

**Figure 3:**
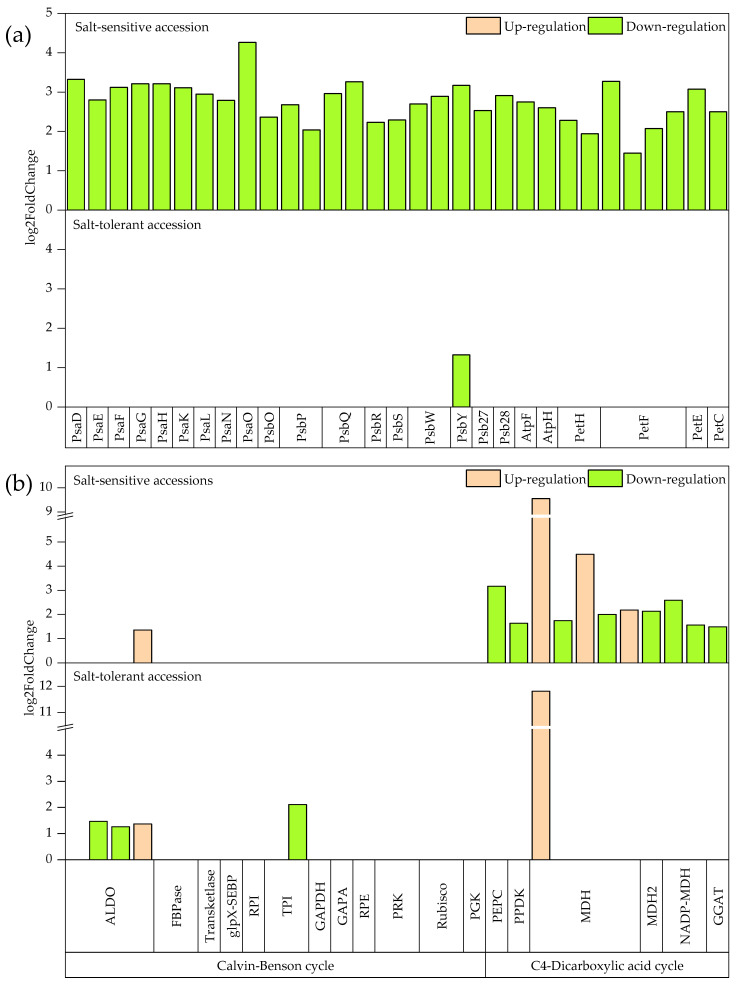
Differentially expressed genes in photosynthesis pathway (sita00195) (**a**) and carbon fixation in photosynthetic pathway (sita00710) (**b**) in salt-sensitive and salt-tolerant accessions. The green and red colors represent down- and up-regulated genes, respectively.

Among the salt-sensitive accession, the photosynthesis pathway was the most affected. In the photosynthesis pathway, the DEGs were divided into five types based on function (Figure 3a, Table S4). For photosystem I, a large number of subunits, such as PsaD, PsaE, PsaF, PsaG, PsaH, PsaK, PsaL, PsaN, and PsaO, were significantly down-regulated only in the salt-sensitive accession (*p* ≤ 0.01). For photosystem II, the genes encoding oxygen-evolving enhancer proteins, such as PsbO, PsbP, and PsbQ, and other protein regulatory genes related to PS II function, such as PsbR, PsbS, PsbW, PsbY, Psb27, and Psb28, were significantly decreased in the salt-sensitive accession; however, only PsbY was significantly decreased in the salt-tolerant accession. In F-type ATPase, genes related to photosynthetic electron transport (PET) and cytochrome b6/f complexes, 2, 7, and 1 were down-regulated only in the salt-sensitive accession.

The carbon fixation in the photosynthetic pathway had the greatest change among the pathways enriched in the salt-sensitive and salt-tolerant accessions. This pathway can be divided into the Calvin–Benson cycle and C_4_–dicarboxylic acid cycle. Down-regulated DEGs related to FBPase, transketolase, glpX-SEBP, RPI, GAPDH, RPE, PRK, Rubisco, and PGK were unique in the Calvin–Benson cycle of the salt-sensitive accession (Figure 3b, Table S4). The same DEGs related to fructose-bisphosphate aldolase (FBA) were found in both accessions and the degree of change of common DEGs in the salt-sensitive accession was more significant than those in the salt-tolerant accession. In the C_4_–dicarboxylic acid cycle, DEGs related to MDH and TPI were detected in both accessions, and the differential expression value of DEGs related to MDH was very high. The down-regulated DEGs related to PEPC, PPDK, GGAT, MDH2, and NADP-MDH were identified only in the salt-sensitive accession.

### 2.6. Transcription Factors (TFs) in Response to Salt Stress

**Figure 4:**
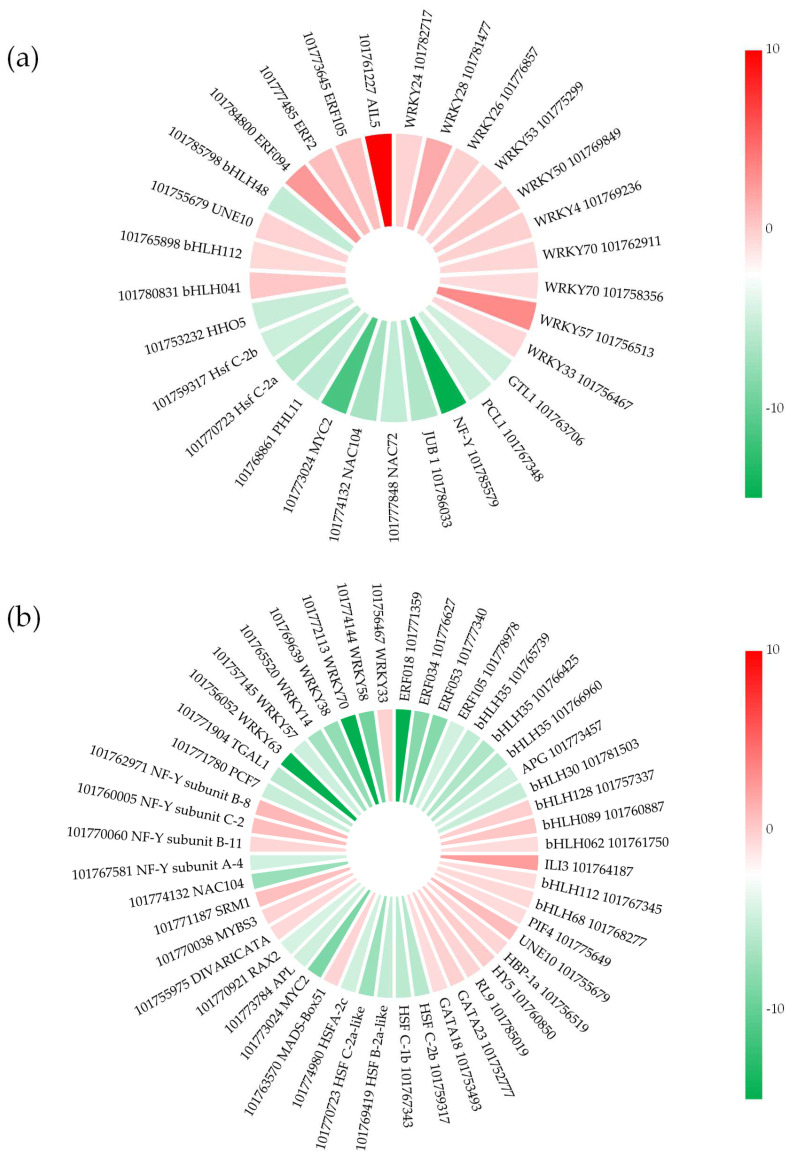
Differential expression of transcription factor salt-sensitive (**a**) and salt-tolerant (**b**) accessions. Color key indicates the log_2_FoldChange.

Salt stress-induced genes are regulated by stress response mechanisms, some of which are regulated by transcription factors (TFs). In this study, 71 TFs were significantly enriched through the Plant TFDB database (Figure 4, Table S5). According to sequence characteristics, the differentially expressed TFs were classified into 15 TF families. Among them, the bHLH and WRKY TF families comprised the largest number of DEGs, followed by the AP2/ERF, MYB-MYC, HSF, and NF TF families. We found 13 families of differentially expressed TF genes in the salt-sensitive accession, of which 21 genes were down-regulated and 27 were up-regulated. In addition, 10 families of differentially expressed TF genes were detected in the salt-tolerant accession, of which 16 genes were down-regulated and 13 were up-regulated. There were six differentially expressed TFs in the two accessions. In the salt-sensitive accession, the response to salt stress was regulated by increasing the number of down-regulated genes of the AP2/REF, HSF, and WRKY families and up-regulated genes of the GATA family. However, in the salt-tolerant accession, a number of coding genes of the AP2-ERE and WRKY families were mainly down-regulated and that of the NAC family mainly up-regulated. In addition, a large number of DEGs of the bHLH and MYB-MYC families were found in the salt-sensitive accession. 

### 2.7. Protein–Protein Interaction Networks in Response to Salt Treatments

**Figure 5:**
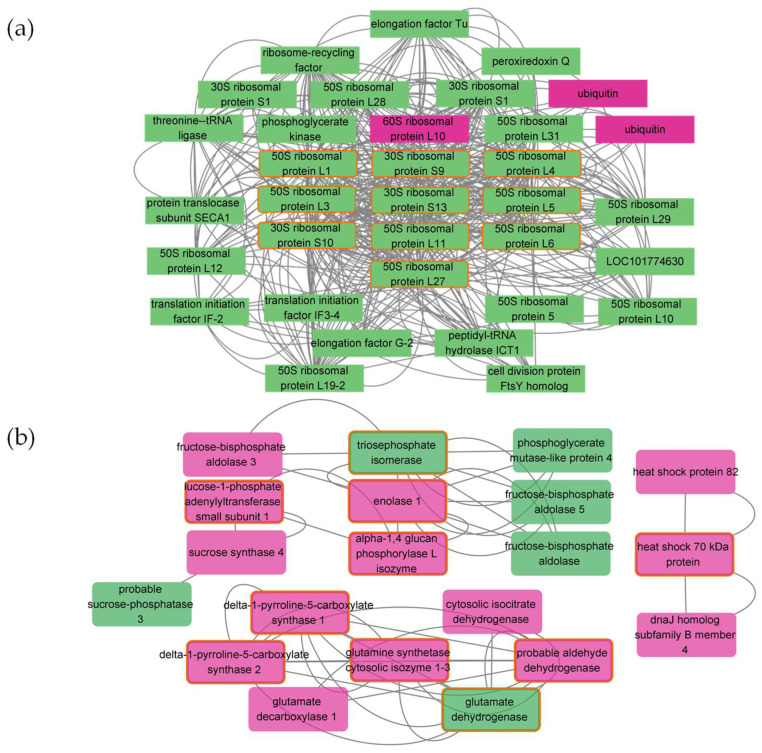
Protein–protein interaction network of Hub genes in salt-sensitive (**a**) and salt-tolerant (**b**) accessions. The green and red colors represent down- and up-regulated genes, respectively. All interactions had the highest confidence (0.900) to minimize false functional links.

To identify master regulators of salt defense-related genes, the protein–protein interaction (PPI) network was utilized to screen key proteins related to salt defense in foxtail millet. Based on the STRING database, 2232 and 68 protein pairs were obtained in the comparison groups of the salt-sensitive (Figure 5a, Table 2) and salt-tolerant (Figure 5b, Table 2) accessions, respectively. For the salt-tolerant accession, a large number of nodes were concentrated on photosynthesis or related regulation networks. Hub genes were also characterized using the CytoHubba. For the salt-sensitive accession, all 10 hub genes were related to ribosomal proteins (Figure 5a, Table 2). For the salt-tolerant accession, the five highest-scoring hub genes were associated with the synthesis of proline and glutamate, but these genes were also enriched in the salt-sensitive accession (Figure 5b, Table 2). Four hub genes annotated to triosephosphate isomerase, enolase 1, alpha-1,4 glucan phosphorylase L isozyme, and glucose-1-phosphate adenylyltransferase small subunit 1 were found only in the salt-tolerant accession.

## 3. Discussion

Plant genetic resources are the basis of cultivar breeding. Different varieties of crops harbor various traits, accumulated due to natural and human factors [23,24]. The key to improving and protecting original varieties and breeding new ones is to identify optimal genetic resources [19,25]. Seed germination is the key period in establishing foxtail millet under salt stress due to water requirements for seed imbibition and germination [26]. Germination rate, germination potential, germination index, plumule length, and radicle length are the most basic indices of salt tolerance in different genetic resources [27,28,29]. The low mean values and large variation coefficients for RGR, RGP, RGI, RPL, and RRL indicate a significant inhibition of seed vigor and that extensive variations probably occurred in salt tolerance in foxtail millet accessions (Table S1). The lowest median plumule and radicle lengths also indicated that their growths were more sensitive to salt. Crops are susceptible to salt stress at the seedling stage because salt toxicity and osmotic stress prevent plant uptake of mineral elements and synthesis of organic matter [30]. Secondary salt-tolerance identification at the seedling stage for foxtail millet initially selected on the basis of the germination test can more accurately identify the salt tolerance of the crop (Table 1 and Table S2). The FM6 accession was the highest comprehensive rank in salt tolerance at the seedling stage, and its salt tolerance rank increased with increasing salt concentration. FM90, the lowest comprehensive rank, was susceptible to all concentrations of salt stress. 

The molecular mechanism underlying the responses to salt stress could provide important clues for further study and in-depth characterization of salt-resistance candidate genes in crops [31]. Recently, the development of novel high-throughput sequencing has provided allowed salt-related genes in different accessions to be identified via de novo assembly or mapping, thereby elucidating the molecular mechanisms underlying plant responses to salt [30]. Recently, large amounts of genomic and transcriptomic data have been obtained for both model and non-model organisms, including rice [32], Kentucky bluegrass [33], and barley [34]. These studies provided deeper insights into the salt tolerance mechanism in crops and accelerated the breeding of a salt-tolerant line [34,35]. With the completion of the sequencing of the foxtail millet genome, the investigation of the transcriptome of foxtail millet has gradually increased. In the PEG stress test in foxtail millet’s germination stage [36], the categories related to phenylpropanoid biosynthesis, plant hormone signal transduction, and phenylalanine metabolism were highly enriched via effects on the phenylpropanoids-related pathway regulating allelochemicals for adaptation. In two transcriptome analyses of foxtail millet at the seedling stage with different drought tolerances [37], chlorophyll metabolism, reactive oxygen species system, abscisic acid metabolism changes, and organic solutes metabolism were some of the important biological pathways in response to drought stress. However, the transcriptome in response to salt in foxtail millet leaf is limited. Here, the most salt-tolerant foxtail millet accession, FM6, and the most salt-sensitive foxtail millet accession, FM90 (Table 1), were chosen for transcriptome sequencing to identify genes conferring salt-tolerance. The percentage of clean reads was above 99%. We mapped more than 90% of reads to foxtail millet genome annotations and, of the total mapped reads, 93% were uniquely mapped (Table S6) [38,39]. The DEGs’ number in the salt-tolerant accession was much lower than that in the salt-sensitive accession.

The DEGs were classified by functional annotation and enrichment analysis into biological process, molecular function, and cellular component. The degree of change of the DEGs located in the biological process and cell component was much higher in the salt-sensitive accession than in the salt-tolerant accession (Figure 1), indicating that the salt-sensitive accession was more affected by biological function and gene product sites or action sites. The photosynthetic capacity was the major process affected by salt stress in the salt-sensitive accession, and the most enriched terms of annotated DEGs in the salt-sensitive accession were localized to the cell and were related to plastids, chloroplasts, and thylakoids, suggesting that the function of these organelles in the salt-sensitive accession was disrupted under salt stress. These results were similar to those observed by Do Amaral et al. [40] in rice and were consistent with the lower leaf chlorophyll content and more intense leaf yellowing in the salt-sensitive millet accession compared with those in the salt-tolerant accession [41,42]. The phosphorus metabolism of the salt-tolerant accession and the terms of the membrane system were significantly enriched under salt stress, which may be attributed to the ability of the salt-tolerant accession to increase salt tolerance by improving the stability of the membrane system [43]. Similar to the GO functional enrichment, the number of enriched KEGG pathways and the number and fold change of DEGs with the KEGG pathways was higher in the salt-sensitive accession than in the salt-tolerant accession, particularly in relation to the photosynthetic term (Figure 3, Table S3). Previous transcriptome studies showed that the genes related to PS II oxygen-evolving enhancer protein function and subunit function in PS I decreased significantly under salt stress [44,45]. In our study, those genes were down-regulated in foxtail millet leaves under salt stress in the salt-sensitive accession (Figure 3), which indicated that this accession was stressed mainly by light absorption [46,47]. The decrease in light absorption capacity further led to decreased F-type ATPase and PET in the salt-sensitive accession [48,49]. Notably, a similar phenomenon was found in the drought-sensitive accession of pearl millet under drought [50], which may imply that the light absorption capacity of millet with poor stress resistance is the most sensitive. The decrease in light absorption capacity directly affected the carbon fixation of salt-sensitive foxtail millet accession [47,51]. We observed that the genes related to Calvin–Benson cycle enzymes and C_4_–dicarboxylic acid cycle enzymes were also significantly down-regulated in the salt-sensitive accession (Figure 3, Table S4). 

TFs are essential upstream regulatory proteins of genes and play an important role in a plant’s response to abiotic stress [52]. Under more severe salt stress, more differentially expressed TFs are detected in the salt-sensitive accession [18,53]. Only six TFs were significantly different between the salt-sensitive and salt-tolerant accessions (Figure 4, Table S5). Among them, the TFs of the bHLH, WRKY, and MYB-MYC families retained a large number of DEGs and followed different patterns of differential expression in the two accessions, which was an important factor contributing to the difference in salt tolerance in foxtail millet. The WRKY family was widely involved in plant responses to stress and was the most abundantly expressed TF family in this study. Eight TFs found only in the salt-tolerant accession had been identified under salt stress in other crops: WRKY4 [54], WRKY24 [55], WRKY26 [56], WRKY28 [55], WRKY50 [57], WRKY53 [58], WRKY57 [59], and WRKY70 [55], and these may be critical in influencing salt tolerance in foxtail millet. AP2/ERF family TFs play a regulatory role in abiotic stress response and could affect growth and metabolism. In this study, four DEGs of the AP2/ERF TF family, AIL5, ERF2, ERF105, and ERF094, were up-regulated in the salt-tolerant accession, and four DEGs of the AP2/ERF family, ERF018, ERF034, ERF053 and ERF105, were down-regulated in the salt-sensitive accession. ERF2 is involved in phytohormone signal cascades [60] and has been identified as a salt-induced target of miRNAs in previous studies [61]. It is generally believed that ERF105 is a cold-regulated TF, but it has also been reported to play a role in plant salt stress, such as down-regulation in creeping bentgrass (*Agrostis stolonifera*) [62] and up-regulation in *Arabidopsis thaliana*. In this study, the completely different expression of ERF105 in the two germplasms suggests that ERF105 may be the key to the ability of salt-tolerant germplasms to cope with salt stress. AIL5 and ERF094 were found less frequently in other plants under salt stress, and the mechanism needs to be studied further in the future. In addition, although the TFs of the HSF family were related to plant heat tolerance, the high degree of enrichment showed that a part of the metabolic pathway in response to heat stress could also regulate the salt tolerance in foxtail millet. These results provide new insights into the salt tolerance mechanism of foxtail millet and will facilitate salt-tolerance breeding in future research.

Ribosomal proteins are important components of the ribosome and have a significant impact on its transcriptional efficiency and stability. In addition, they are involved in various important intracellular activities, such as DNA repair, apoptosis, and regulation of gene expression. In the salt-sensitive accession, all 10 hub genes were related to ribosomal proteins and all were down-regulated, which may cause stunted growth in foxtail millet plants (Figure 5a). The salt-tolerant accession was annotated with fewer proteins, and their protein–protein interactions were weak due to less stress. Among the hub genes in the salt-tolerant accession, the four genes expressed only in the salt-sensitive accession were associated with the catabolism and synthesis of sugars, suggesting that salt-tolerant foxtail millet accession can catabolize to produce more energy under salt stress (Figure 5b). The relationship between genes (101760718 and 101781167) related to triosephosphate isomerase and enolase 1 and salt stress has been reported by Chen et al. [63] and Nam et al. [64]. However, to our knowledge, the response of alpha-1,4 glucan phosphorylase L isozyme (101773221) and glucose-1-phosphate adenylyltransferase small subunit 1 (101755337) has not been reported and requires further study (Table 2).

## 4. Materials and Methods

### 4.1. Plant Materials and Growth Conditions

The salt tolerance test of 104 foxtail millet accessions of China (Table S7) was performed to screen for salt-tolerant and salt-sensitive foxtail millet accessions. Specifically, 20 foxtail millet seeds were sterilized with 75% ethanol and germinated in a sterile culture dish (10 × 10 × 2 cm) containing 50 mL Murashige and Skoog (MS) solid medium containing 0.17 mol·L^−1^ NaCl (0.17 mol·L^−1^ NaCl was used because the maximum coefficient of variation was found for 104 foxtail millet accessions at this concentration in the pre-experiment). All culture tests were conducted in triplicates. Germination was considered when either the plumule reached half the length of the seed or the radicle reached the length of the seed. Germination was recorded every 2 days and, after 8 days, the germination rate (GR), plumule length (PL), and radicle length (RL) were measured. The germination potential (GP) and germination index (GI) represent seed vigor. The GP was the germination rate on the 4th day. The GI was calculated as follows:

(1) $$\mathrm{GI}=\sum \frac{{\mathrm{GR}}_{t}}{D_{t}}$$

where GR_t_ is the GR on t day and D_t_ is the treatment days. GR, GP, GI, PL, and RL describe the seed germination process. To show seed changes under salt stress, relative GR (RGR), relative GP (RGP), relative GI (RGI), relative PL (RPL), and RL (RRL) were determined as the quotient of traits under salt stress versus salt-free conditions. The membership function method in fuzzy mathematics was used for the preliminary evaluation of the salt-tolerant and salt-sensitive accessions as it compensates for the bias caused by a single indicator. The membership function was calculated as follows:

(2) $$X_{\left(\mathrm{ij}\right)}=\left(X_{\mathrm{ij}}-X_{\mathrm{jmin}}\right)/\left(X_{\mathrm{jmax}}-X_{\mathrm{jmin}}\right)$$

where X_ij_ is the measured value of index j of category i; X_jmin_ and X_jmax_ are the maximum and minimum values of index j of category i, respectively; and X_(ij)_ is the membership value of index j of category i.

The salt tolerance test of the foxtail millet at the seedling stage was conducted to screen the potentially most salt-tolerant and salt-sensitive accessions. Seeds were sterilized with 75% ethanol and cultured on the MS solid medium. After 8 days, 50 seedlings were transplanted into a plastic pot (diameter 30 cm, height 20 cm) with sterile sand (particle size ≤ 1 mm) and watered with 100 mL of 1/2 Hoagland nutrient daily. After 12 days, 100 mL of 1/2 Hoagland nutrient containing 0.13 mol·L^−1^, 0.17 mol·L^−1^, and 0.22 mol·L^−1^ NaCl was added daily. The foxtail millet seedlings were continuously watered with saline nutrient solution for 6 days. The relative biomass, relative growth rate, relative chlorophyll content, and relative K^+^/Na^+^ of the leaves were measured. The growth rate was calculated as the average increase in plant height per day. For biomass, 10 seedlings were randomly selected, washed with distilled water, and weighed using a one hundred thousandth scale. For the chlorophyll content, the supernatant of the fresh leaf samples soaked in dimethyl sulfoxide under dark condition for 3 days was measured using a spectrophotometer at 663 and 645 nm [14]. For Na^+^ and K^+^ contents of the leaves, 0.1 g dry powder of leaf samples (≤0.25 mm) was digested with a mixture of concentrated HNO_3_ and HClO_4_ (2:1 V:V) at 370 °C for 3 h, and the contents were then determined via flame photometer (AP1500, AoPu, Shanghai, China) [14,65]. The membership function method in fuzzy mathematics was used to evaluate the performance of different foxtail millet accessions under salt stress. Finally, the most promising salt-tolerant and salt-sensitive foxtail accessions were confirmed. All plant culture experiments were performed in an artificial growth chamber (Adaptis-A1000; CONVIRON, Winnipeg, Canada) at 30 ± 1 °C day/20 ± 1 °C night temperatures, with a 16/8 h (day/night) photoperiod and 500 μmol m^−2^ s^−1^ PPFD.

### 4.2. Treatments and Experimental Design

Based on the salt tolerance evaluation, the most salt-tolerant (Hong Gu 2000) and salt-sensitive (Pu Huang Yu) accessions were obtained from 104 foxtail millet accessions. According to the culture method of the salt tolerance test of foxtail millet seedling, the most salt-sensitive and salt-tolerant accessions were sampled after exposure to 0.17 mol·L^−1^ NaCl stress for 6 days and were labeled SS and TS, respectively. Leaf samples were also sampled from the salt-sensitive and salt-tolerant accessions treated with unsalted Hoagland nutrient solution and named SC and TC, respectively. There were three replicates for each treatment, and all leaves from 12 samples were individually frozen using liquid nitrogen and stored at −80 °C in preparation for RNA-Seq analysis.

### 4.3. RNA Isolation, cDNA Library Preparation, and Sequencing

The total RNA of foxtail millet leaves was extracted using the Trizol method (1 mL of Trizol reagent was added into 0.1 g of leaf sample) [66]. The concentrations and purity were measured using a NanoDrop 2000 spectrophotometer (Thermo Fisher Scientific, Wilmington, DE, USA). The integrity of RNA samples was determined using the Agilent Bioanalyzer 2100 System (Agilent Technologies, Palo Alto, CA, USA) and all the RNA Integrity Number values were above 8. mRNA with poly-A structure was enriched with oligo magnetic beads in the total RNA and then cut into 200–300 bp fragments. Using these RNA fragments as a template, we synthesized the first chain of cDNA with random hexamers and reverse transcriptase. This first chain cDNA was then used for the synthesis of the second chain cDNA. The library fragments were enriched using PCR, and the obtained 300–400 bp libraries were selected and tested with an Agilent 2100 Bioanalyzer (Santa Clara, California, United States). The RNA library was constructed using Illumina HiSeq™ 2000 (Illumina, San Diego, CA, USA) at the Shanghai Personal Biotechnology Co., Ltd., China (http://www.personalbio.cn/ (accessed on 18 December 2018)).

### 4.4. Bioinformatics Analysis

For the original raw off-machine data, Cutadapt was used to remove the adaptor of the 3′-end (the removed part contained a mismatch of at least 10 bp overlap with a known connector, an approximately 20% base mismatch) and the low-quality reads (QV < 20) [67]. Transcriptomic alignments were identified using Bowtie2 (University of Maryland, College Park, MD, USA) [68,69] and Tophat2 (Johns Hopkins University, Maryland, USA) [70,71] against the foxtail millet reference genome (GCF_000263155.2_Setaria_italica_v2.0_genomic.fa) (Table S6), which was downloaded from the Ensembl database [72]. The mapping criteria were as follows: sequencing reads should be uniquely matched to the genome while allowing up to two mismatches, without insertions or deletions. The HTSeq and union models were used to analyze the gene expression. DESeq (http://www.bioconductor.org/packages/release/bioc/html/DESeq.html (accessed on 18 December 2018)) was used to compare the read count value on each gene as the original gene expression amount, and then the fragments per kilobase of transcript per million mapped reads (FPKM) method was adopted to standardize the expression amount [73]. Genes with a |log_2_FoldChange| > 1 and significant difference at *p* < 0.01 were considered DEGs.

The DEGs were submitted for functional enrichment analyses of gene ontology (GO), Kyoto Encyclopedia of Genes and Genomes (KEGG) pathways, and transcription factor (TF) [74]. GOseq software (http://www.bioconductor.org/packages/release/bioc/html/goseq.html (accessed on 8 December 2018)) was used to perform GO analysis with the default parameters and *p*-value < 0.01 [75]. KEGG [76,77] enrichment analyses were performed using KOBAS software (version 3.0) (http://kobas.cbi.pku.edu.cn/download.php) with the default parameters and *p*-value < 0.01 [78]. GO terms and KEGG terms with corrected *p*-values less than 0.01 were considered significantly enriched. TF were DEGs with TF function annotated to the reference genome. STRING (http://www.string-db.org/ (accessed on 18 December 2018)) was applied to protein–protein interaction analysis of protein function, protein subcellular localization, and gene co-expression using Linum usitatissimum as the standard [18,79]. The PPI network was constructed according to the highest confidence level (0.900) in STRING [80,81]. Cytoscape (version: 3.9.1) [82,83] and Cytohubba [84] were used to calculate 10 DEGs as hub genes by maximal clique centrality (MCC) and display all DEGs associated with the hub gene, which were then annotated with NCBI blast.

## 5. Conclusions

Salt-tolerant (Hong Gu 2000) and salt-sensitive (Pu Huang Yu) accessions were identified among 104 foxtail millet accessions during the germination and seedling stages, using morpho-physiological indicators based on the membership function method in fuzzy mathematics. A large-scale transcriptome dataset in foxtail millet in response to salt stress was established. Through analyzing the differences in the transcriptome of foxtail millet leaves under 0.17 mol·L^−1^ NaCl stress, 2019 and 736 DEGs were obtained in the salt-sensitive and salt-tolerant accessions, respectively. Most differential genes in the salt-sensitive accession were concentrated in plastids, chloroplasts, and thylakoids, and the photoabsorption and carbon fixation capacities decreased significantly. The down-regulation of ribosomal protein-related genes inhibited the growth of salt-sensitive varieties. Salt-tolerant varieties alleviate salt stress by increasing glucose and ATP production. There were significant differences in the expression of transcription factors between the salt-tolerant and salt-sensitive accessions. Most of these TFs were concentrated in the BHLH, WRKY, AP2/ERF, and MYB-MYC families, which significantly affected the salt-tolerance of the two foxtail millet accessions. These findings provide novel insights into the molecular mechanism underlying foxtail millet’s response to salt stress.

## Figures and Tables

**Table 1:** Comprehensive evaluation of salt tolerance of 18 foxtail millet accessions in seeding foxtail millet under different NaCl concentrations. The score and rank under different NaCl concentrations indicate the scores and rank at that concentration, while the total score and rank indicate the comprehensive scores and rank under all NaCl concentrations.

Code	0.13 Mol·L^−1^		0.17 Mol·L^−1^		0.22 Mol·L^−1^		Total Score	Total Rank
	Score	Rank	Score	Rank	Score	Rank		
FM6	3.63	3	3.31	2	3.18	1	10.12	1
FM7	3.49	4	2.74	4	2.50	5	8.74	4
FM31	3.38	5	2.11	5	2.62	4	8.12	5
FM96	3.85	1	3.41	1	2.75	3	10.02	2
FM73	3.69	2	2.95	3	2.89	2	9.53	3
FM15	0.76	13	0.28	17	0.43	12	1.48	16
FM27	1.89	7	0.66	9	0.56	11	3.11	7
FM41	0.50	17	0.41	15	0.41	15	1.33	17
FM48	0.53	16	0.57	10	0.67	7	1.77	13
FM49	0.79	11	0.50	11	0.60	8	1.88	11
FM62	1.19	8	0.86	6	0.71	9	2.77	8
FM66	0.56	15	0.40	13	0.95	6	1.91	10
FM72	0.77	12	0.41	14	0.45	17	1.62	15
FM78	2.09	6	0.77	7	0.42	16	3.27	6
FM86	1.08	9	0.48	12	0.48	13	2.03	9
FM89	0.90	10	0.38	16	0.48	10	1.76	14
FM90	0.47	18	0.26	18	0.14	18	0.87	18
FM102	0.74	14	0.67	8	0.45	14	1.87	12

**Table 2:** Hub gene information by maximal clique centrality method.

Treatment	Rank	Gene Symbol	Gene Description	Score
SS/SC	1	LOC101773632	30S ribosomal protein S9	6.28 × 10^9^
	2	LOC101762479	S ribosomal protein L3	6.28 × 10^9^
	3	LOC101757542	50S ribosomal protein L1	6.28 × 10^9^
	4	LOC101761760	30S ribosomal protein S10 X2	6.28 × 10^9^
	5	LOC101778257	50S ribosomal protein L4	6.28 × 10^9^
	6	LOC101771094	50S ribosomal protein L5	6.28 × 10^9^
	7	LOC101777693	30S ribosomal protein S13	6.28 × 10^9^
	8	LOC101780142	50S ribosomal protein L11	6.28 × 10^9^
	9	LOC101780276	50S ribosomal protein L6	6.28 × 10^9^
	10	LOC101776379	50S ribosomal protein L27	6.28 × 10^9^
TS/TC	1	LOC101770922	probable aldehyde dehydrogenase	25
	1	LOC101778373	glutamate dehydrogenase	25
	3	LOC101754854	glutamine synthetase cytosolic isozyme 1-3	24
	3	LOC101775420	delta-1-pyrroline-5-carboxylate synthase 2	24
	3	LOC101765114	delta-1-pyrroline-5-carboxylate synthase 1	24
	6	LOC101781167	enolase 1, chloroplastic	9
	7	LOC101760718	triosephosphate isomerase, cytosolic	8
	8	LOC101755337	glucose-1-phosphate adenylyltransferase small subunit 1, chloroplastic/amyloplastic	2
	8	LOC101773221	alpha-1,4 glucan phosphorylase L isozyme, chloroplastic/amyloplastic	2
	8	LOC101756252	heat shock 70 kDa protein	2

## Data Availability

The datasets used in this study can be found in the NCBI SRA database under the accession number PRJNA805389.

## Supplementary Materials

The following supporting information can be downloaded at: https://www.mdpi.com/article/10.3390/plants11141864/s1, Table S1. Salt tolerance identification of 104 foxtail millet accessions in germination stage under 0.13 mol L-1. Table S2. Comprehensive evaluation of salt tolerance of 18 foxtail millet accessions in seeding foxtail millet under different NaCl concentrations. The score and rank under different NaCl concentrations indicate the scores and rank at that concentration, while the total score and rank indicate the comprehensive scores and rank under all NaCl concentrations. Table S3. Enrichment analysis of KEGG between SS/SC and TS/TC. Table S4. DGEs associated with photosynthesis under salt stress. Table S5. DEGs related to transcription factor under salt stress. Table S6. Sequencing statistics of RNA libraries. Table S7. 104 foxtail millet accessions for testing in the study.
