# Supplementary material for: Transcriptome Analysis Reveals Molecular Mechanisms under Salt Stress in Leaves of Foxtail Millet (Setaria italica L.)
Source: Plants (Basel). 2022 Jul 18;11(14):1864. doi: 10.3390/plants11141864 (PMC9323065; doi:10.3390/plants11141864)
Supplement: Supplementary file 1 [file plants-11-01864-s001.zip › plants-1777431-Table S1.pdf]

Table S1. Salt tolerance identification of 104 foxtail millet accessions in germination stage under 0.13 mol L^{-1}

Code	Relative germination rate	Relative germination potential	Relative germination index	Relative plumule length	Relative root length	Score	Rank
FM1	33.33%	29.41%	27.72%	5.81%	0.00%	1.45	72
FM2	44.44%	43.75%	50.00%	39.52%	8.84%	2.85	23
FM3	30.77%	33.33%	33.91%	3.60%	4.40%	1.61	68
FM4	20.00%	12.50%	19.28%	36.17%	28.57%	1.84	61
FM5	35.00%	25.00%	22.07%	11.43%	22.86%	1.80	63
FM6	56.25%	60.00%	52.17%	48.49%	49.76%	4.12	4
FM7	42.86%	75.00%	50.00%	41.78%	52.85%	4.03	5
FM8	50.00%	35.00%	39.17%	39.98%	42.82%	3.23	12
FM9	56.25%	53.33%	52.81%	45.45%	39.02%	3.81	7
FM10	20.00%	20.00%	20.00%	3.42%	0.00%	0.95	85
FM11	50.00%	46.15%	47.06%	10.45%	8.37%	2.46	38
FM12	60.00%	45.00%	47.08%	48.09%	37.61%	3.68	8
FM13	55.00%	35.00%	38.89%	23.63%	25.91%	2.75	27
FM14	31.58%	26.32%	25.00%	31.32%	20.85%	2.08	53
FM15	0.00%	0.00%	0.00%	0.00%	0.00%	0.00	92
FM16	45.00%	40.00%	36.67%	7.22%	25.14%	2.37	41
FM17	12.50%	12.50%	14.29%	17.81%	33.53%	1.44	73
FM18	31.58%	31.58%	27.78%	8.10%	18.71%	1.81	62
FM19	15.38%	18.18%	17.91%	3.80%	0.00%	0.83	87
FM20	66.67%	47.06%	46.07%	5.50%	20.25%	2.83	24
FM21	33.33%	29.41%	31.52%	2.72%	18.22%	1.78	64
FM22	47.37%	31.58%	37.50%	4.01%	2.73%	1.87	58
FM23	23.53%	18.75%	17.89%	4.33%	0.00%	0.97	84
FM24	44.44%	50.00%	47.37%	45.07%	22.22%	3.20	13
FM25	38.89%	46.67%	56.60%	9.52%	11.28%	2.50	36
FM26	47.06%	57.14%	39.29%	6.40%	0.00%	2.23	46
FM27	0.00%	0.00%	0.00%	0.00%	0.00%	0.00	93
FM28	33.33%	27.78%	34.41%	8.22%	32.76%	2.14	51
FM29	35.29%	35.29%	30.30%	4.94%	0.00%	1.58	70
FM30	11.11%	11.76%	11.54%	3.15%	0.00%	0.57	91
FM31	61.11%	50.00%	56.91%	62.25%	48.73%	4.33	3
FM32	35.29%	46.15%	35.09%	10.55%	18.49%	2.21	47
FM33	35.29%	33.33%	32.04%	5.15%	9.62%	1.76	65
FM34	62.50%	53.85%	51.23%	7.12%	3.99%	2.69	29

FM35	38.89%	35.29%	40.00%	15.09%	32.78%	2.52	34
FM36	50.00%	52.63%	41.38%	10.48%	43.55%	3.06	18
FM37	43.75%	41.67%	38.46%	11.94%	8.84%	2.19	49
FM38	65.00%	65.00%	50.00%	5.53%	12.20%	2.98	20
FM39	17.65%	17.65%	20.00%	4.22%	0.00%	0.90	86
FM40	36.84%	33.33%	33.65%	42.23%	49.71%	3.07	17
FM41	0.00%	0.00%	0.00%	0.00%	0.00%	0.00	94
FM42	46.15%	46.15%	44.00%	52.50%	10.18%	3.03	19
FM43	45.00%	40.00%	31.25%	56.43%	50.79%	3.47	10
FM44	35.00%	21.05%	27.27%	7.65%	8.31%	1.52	71
FM45	50.00%	33.33%	34.31%	11.21%	8.26%	2.08	52
FM46	52.63%	42.11%	38.74%	8.77%	15.15%	2.40	40
FM47	31.58%	22.22%	25.70%	5.10%	5.89%	1.38	74
FM48	0.00%	0.00%	0.00%	0.00%	0.00%	0.00	95
FM49	0.00%	0.00%	0.00%	0.00%	0.00%	0.00	96
FM50	15.79%	10.53%	12.28%	5.37%	7.19%	0.79	89
FM51	50.00%	50.00%	51.72%	19.35%	30.67%	3.11	16
FM52	45.00%	35.00%	35.83%	28.75%	16.82%	2.47	37
FM53	50.00%	44.44%	44.76%	34.48%	17.51%	2.93	22
FM54	15.00%	12.50%	14.29%	4.62%	2.19%	0.74	90
FM55	56.25%	66.67%	59.59%	8.90%	30.78%	3.40	11
FM56	50.00%	38.89%	43.62%	3.56%	43.26%	2.80	25
FM57	50.00%	50.00%	52.94%	4.27%	17.22%	2.67	30
FM58	45.00%	33.33%	35.38%	30.48%	22.58%	2.57	32
FM59	66.67%	44.44%	48.15%	14.29%	18.63%	2.94	21
FM60	28.57%	36.36%	33.33%	5.16%	2.70%	1.60	69
FM61	50.00%	46.67%	47.83%	12.79%	24.59%	2.79	26
FM62	0.00%	0.00%	0.00%	0.00%	0.00%	0.00	97
FM63	50.00%	33.33%	36.67%	24.03%	21.65%	2.55	33
FM64	38.89%	27.78%	31.02%	35.67%	16.42%	2.31	43
FM65	37.50%	37.50%	37.50%	4.27%	4.88%	1.84	60
FM66	0.00%	0.00%	0.00%	0.00%	0.00%	0.00	98
FM67	28.57%	27.27%	28.99%	4.20%	0.00%	1.34	76
FM68	55.00%	46.67%	47.65%	41.96%	40.97%	3.60	9
FM69	42.11%	41.18%	42.72%	41.18%	35.81%	3.15	14
FM70	38.89%	35.29%	31.19%	13.29%	17.58%	2.08	54
FM71	47.06%	53.85%	51.16%	53.91%	44.93%	3.88	6
FM72	0.00%	0.00%	0.00%	0.00%	0.00%	0.00	99

FM73	63.16%	58.82%	58.29%	65.00%	40.40%	4.40	1
FM74	15.38%	25.00%	21.24%	3.13%	4.69%	1.05	82
FM75	25.00%	30.00%	44.35%	28.57%	11.11%	2.15	50
FM76	50.00%	37.50%	30.93%	6.06%	0.00%	1.86	59
FM77	35.29%	28.57%	26.51%	40.45%	15.04%	2.24	45
FM78	0.00%	0.00%	0.00%	0.00%	0.00%	0.00	100
FM79	47.06%	50.00%	43.82%	42.86%	20.00%	3.11	15
FM80	6.25%	7.14%	8.22%	41.04%	12.00%	1.16	81
FM81	25.00%	20.00%	12.86%	14.84%	16.63%	1.37	75
FM82	26.32%	22.22%	21.63%	9.04%	8.26%	1.34	77
FM83	21.05%	25.00%	21.99%	33.63%	46.15%	2.33	42
FM84	25.00%	30.00%	28.13%	2.05%	1.45%	1.30	78
FM85	30.00%	25.00%	26.67%	8.06%	17.70%	1.66	67
FM86	0.00%	0.00%	0.00%	0.00%	0.00%	0.00	101
FM87	20.00%	21.05%	18.10%	36.36%	44.52%	2.21	48
FM88	30.00%	30.00%	25.00%	41.67%	28.57%	2.40	39
FM89	0.00%	0.00%	0.00%	0.00%	0.00%	0.00	102
FM90	0.00%	0.00%	0.00%	0.00%	0.00%	0.00	103
FM91	15.79%	11.11%	8.21%	27.24%	15.53%	1.21	80
FM92	45.00%	20.00%	29.52%	9.60%	8.95%	1.74	66
FM93	40.00%	38.89%	37.23%	37.34%	19.78%	2.66	31
FM94	33.33%	33.33%	28.57%	26.50%	13.39%	2.06	55
FM95	35.00%	10.53%	18.32%	1.97%	1.72%	1.03	83
FM96	63.16%	66.67%	45.65%	50.67%	58.14%	4.38	2
FM97	21.05%	11.11%	9.90%	5.26%	5.03%	0.80	88
FM98	43.75%	20.00%	26.32%	12.70%	26.67%	2.02	57
FM99	44.44%	40.00%	35.08%	36.95%	20.67%	2.71	28
FM100	31.58%	11.11%	21.05%	6.84%	10.75%	1.27	79
FM101	55.56%	37.50%	32.94%	22.04%	16.43%	2.51	35
FM102	0.00%	0.00%	0.00%	0.00%	0.00%	0.00	104
FM103	40.00%	30.00%	18.86%	28.41%	15.76%	2.02	56
FM104	47.37%	44.44%	30.23%	19.23%	7.84%	2.24	44
Standard error	1.82	1.76	1.6	1.72	1.54	1.82	-
Mean value	34.36	30.65	29.72	18.12	16.89	34.36	-
Coefficient of variation	53.93	58.42	55.01	96.67	92.88	53.93	-
Maximum	66.67	75	59.59	65	58.14	66.67	-
Minimum	0	0	0	0	0	0	-

Median	35.29	32.46	31.1	9.56	14.22	35.29	-
