# Supplementary material for: Transcriptome Analysis Reveals Molecular Mechanisms under Salt Stress in Leaves of Foxtail Millet (Setaria italica L.)
Source: Plants (Basel). 2022 Jul 18;11(14):1864. doi: 10.3390/plants11141864 (PMC9323065; doi:10.3390/plants11141864)
Supplement: Supplementary file 1 [file plants-11-01864-s001.zip › plants-1777431-Table S2.pdf]

Table S2. Comprehensive evaluation of salt tolerance of 18 foxtail millet accessions in seeding foxtail millet under different NaCl concentrations. The score and rank under different NaCl concentrations indicate the scores and rank at that concentration, while the total score and rank indicate the comprehensive scores and rank under all NaCl concentrations.

Code	0.13 mol L^{-1} NaCl						0.17 mol L^{-1} NaCl						0.22 mol L^{-1} NaCl						Total score	Total rank
	RB	RGR	RCC	RIO	Score	Rank	RB	RGR	RCC	RIO	Score	Rank	RB	RGR	RCC	RIO	Score	Rank		
FM73	86.39% ± 5.37%	83.33% ± 7.34%	83.71% ± 4.38%	89.37% ± 4.15%	3.69	2	69.57% ± 6.01%	62.15% ± 5.72%	58.58% ± 4.74%	56.53% ± 2.92%	2.95	3	38.41% ± 3.02%	30.00% ± 1.84%	37.12% ± 2.42%	41.74% ± 3.24%	2.89	2	9.53	3
FM96	92.94% ± 5.52%	88.76% ± 4.82%	81.36% ± 4.54%	88.88% ± 5.55%	3.85	1	72.78% ± 6.07%	76.78% ± 5.54%	64.39% ± 4.93%	59.68% ± 5.41%	3.41	1	38.43% ± 2.41%	37.64% ± 3.66%	25.81% ± 1.97%	42.25% ± 3.19%	2.75	3	10.02	2
FM31	83.30% ± 3.42%	82.71% ± 7.39%	77.61% ± 3.43%	83.34% ± 5.67%	3.38	5	41.80% ± 3.43%	52.72% ± 4.09%	47.73% ± 4.22%	50.77% ± 2.91%	2.11	5	37.55% ± 2.32%	36.26% ± 2.09%	28.17% ± 1.69%	34.96% ± 2.89%	2.62	4	8.12	5
FM6	87.69% ± 4.24%	93.33% ± 8.76%	78.02% ± 6.61%	81.35% ± 6.31%	3.63	3	80.63% ± 5.01%	70.73% ± 4.58%	52.74% ± 4.13%	65.77% ± 3.97%	3.31	2	41.98% ± 2.83%	37.49% ± 3.65%	32.71% ± 1.31%	50.59% ± 4.55%	3.18	1	10.12	1
FM7	82.35% ± 6.31%	80.44% ± 4.34%	84.09% ± 3.78%	86.15% ± 4.91%	3.49	4	57.94% ± 4.78%	50.81% ± 2.61%	70.11% ± 6.62%	50.31% ± 4.94%	2.74	4	38.01% ± 1.54%	30.13% ± 2.75%	36.28% ± 1.72%	25.43% ± 2.02%	2.50	5	8.74	4
FM102	41.86% ± 2.48%	56.84% ± 3.01%	33.31% ± 1.57%	52.08% ± 2.70%	0.74	14	29.94% ± 2.21%	24.75% ± 1.36%	25.50% ± 1.39%	34.23% ± 2.67%	0.67	8	6.56% ± 0.38%	5.89% ± 0.55%	10.23% ± 0.86%	12.45% ± 0.62%	0.45	14	1.87	12
FM90	42.50% ± 2.54%	54.46% ± 3.34%	22.67% ± 0.96%	49.12% ± 3.93%	0.47	18	23.39% ± 1.12%	22.59% ± 2.01%	28.87% ± 2.25%	24.86% ± 2.01%	0.41	14	4.86% ± 0.28%	3.82% ± 0.25%	6.06% ± 0.44%	7.58% ± 0.54%	0.14	18	1.01	18
FM89	57.82% ± 3.04%	64.71% ± 2.91%	23.66% ± 1.40%	47.34% ± 2.47%	0.90	10	19.56% ± 1.51%	18.04% ± 1.52%	24.76% ± 1.57%	32.13% ± 2.12%	0.38	16	15.68% ± 1.09%	15.27% ± 0.80%	5.78% ± 0.31%	4.81% ± 0.41%	0.48	10	1.76	14
FM86	63.34% ± 3.46%	60.00% ± 2.87%	34.29% ± 2.92%	47.72% ± 2.67%	1.08	9	17.58% ± 1.36%	16.28% ± 0.94%	30.61% ± 1.95%	33.12% ± 2.52%	0.48	12	5.41% ± 0.43%	4.92% ± 0.40%	12.64% ± 1.08%	11.55% ± 0.74%	0.48	13	2.03	9

FM78	80.26% ± 3.52%	78.44% ± 3.87%	59.21% ± 2.87%	46.39% ± 2.56%	2.09	6	27.39% ± 1.47%	25.34% ± 1.29%	30.35% ± 2.05%	34.67% ± 2.79%	0.77	7	4.89% ± 0.41%	3.95% ± 0.33%	12.24% ± 1.03%	10.89% ± 0.82%	0.42	16	3.27	6
FM72	39.32% ± 3.57%	64.06% ± 3.27%	28.35% ± 1.74%	52.29% ± 3.60%	0.77	12	17.60% ± 1.85%	10.90% ± 0.97%	28.25% ± 1.56%	29.61% ± 1.98%	0.26	18	2.21% ± 0.13%	1.97% ± 0.16%	15.07% ± 0.64%	11.78% ± 1.01%	0.45	17	1.48	15
FM66	33.21% ± 1.86%	56.46% ± 4.20%	30.47% ± 1.76%	52.68% ± 5.01%	0.56	15	29.81% ± 2.48%	11.57% ± 0.62%	24.74% ± 2.27%	31.81% ± 1.30%	0.40	13	13.16% ± 1.31%	12.46% ± 1.01%	17.80% ± 1.34%	13.29% ± 0.61%	0.95	6	1.91	10
FM62	63.12% ± 5.51%	67.59% ± 6.50%	28.98% ± 1.91%	50.13% ± 2.16%	1.19	8	38.24% ± 3.27%	34.90% ± 2.10%	23.12% ± 1.81%	33.84% ± 3.03%	0.86	6	5.41% ± 0.53%	4.34% ± 0.35%	18.79% ± 1.56%	14.24% ± 0.90%	0.71	9	2.77	8
FM49	32.67% ± 2.15%	64.80% ± 3.10%	37.69% ± 2.93%	50.74% ± 4.72%	0.79	11	23.13% ± 2.26%	21.74% ± 1.51%	29.09% ± 2.73%	29.01% ± 2.46%	0.50	11	14.08% ± 1.05%	11.37% ± 0.63%	8.59% ± 0.41%	11.48% ± 0.57%	0.60	8	1.88	11
FM48	36.20% ± 2.02%	64.08% ± 4.83%	22.41% ± 0.97%	48.33% ± 2.58%	0.53	16	25.36% ± 2.41%	10.87% ± 0.46%	32.15% ± 2.39%	35.22% ± 3.36%	0.57	10	12.04% ± 1.10%	11.37% ± 0.81%	14.31% ± 1.01%	7.14% ± 0.44%	0.67	7	1.77	13
FM41	43.03% ± 3.60%	42.86% ± 2.65%	32.60% ± 2.56%	53.43% ± 4.13%	0.50	17	19.90% ± 1.85%	16.78% ± 1.29%	27.54% ± 1.28%	31.34% ± 2.43%	0.41	15	5.92% ± 0.58%	5.37% ± 0.38%	10.73% ± 0.78%	10.79% ± 0.58%	0.41	15	1.33	17
FM27	80.11% ± 3.65%	88.33% ± 6.48%	22.13% ± 0.98%	54.96% ± 5.17%	1.89	7	29.29% ± 2.69%	23.13% ± 1.86%	34.57% ± 3.14%	27.28% ± 1.68%	0.66	9	6.40% ± 0.46%	5.02% ± 0.43%	12.15% ± 0.87%	15.60% ± 0.88%	0.56	11	3.11	7
FM15	38.72% ± 1.85%	63.04% ± 5.98%	37.42% ± 2.41%	47.16% ± 2.96%	0.76	13	14.38% ± 2.18%	15.47% ± 1.17%	31.72% ± 2.80%	25.92% ± 1.14%	0.28	17	8.17% ± 0.39%	6.66% ± 0.29%	9.44% ± 0.51%	11.01% ± 1.09%	0.43	12	1.48	16

Note: RB means the relative biomass; RGR means the relative growth rate; RCC means the relative chlorophyll content; RIR means the K^+/Na^+ ratio.
