# Supplementary material for: Transcriptome Analysis Reveals Molecular Mechanisms under Salt Stress in Leaves of Foxtail Millet (Setaria italica L.)
Source: Plants (Basel). 2022 Jul 18;11(14):1864. doi: 10.3390/plants11141864 (PMC9323065; doi:10.3390/plants11141864)
Supplement: Supplementary file 1 [file plants-11-01864-s001.zip › plants-1777431-Table S3.pdf]

Table S3. Enrichment analysis of KEGG between SS/SC and TS/TC

Treatment	Pathway ID	Pathway annotation	Level1	Level2	FDR	P-value
SS/SC	sita00195	Photosynthesis - *Setaria italica*	Metabolism	Energy metabolism	1.53×10^{-19}	1.38×10^{-21}
	sita00710	Carbon fixation in photosynthetic organisms - *Setaria italica*	Metabolism	Energy metabolism	3.04×10^{-12}	5.47×10^{-14}
	sita00500	Starch and sucrose metabolism - *Setaria italica*	Metabolism	Carbohydrate metabolism	3.42×10^{-8}	9.23×10^{-10}
	sita00906	Carotenoid biosynthesis - *Setaria italica*	Metabolism	Metabolism of terpenoids and polyketides	1.76×10^{-7}	6.34×10^{-9}
	sita00630	Glyoxylate and dicarboxylate metabolism - *Setaria italica*	Metabolism	Carbohydrate metabolism	2.38×10^{-5}	1.07×10^{-6}
	sita00250	Alanine, aspartate and glutamate metabolism - *Setaria italica*	Metabolism	Amino acid metabolism	6.31×10^{-5}	3.41×10^{-6}
	sita00960	Tropane, piperidine and pyridine alkaloid biosynthesis - *Setaria italica*	Metabolism	Biosynthesis of other secondary metabolites	1.08×10^{-4}	6.78×10^{-6}
	sita00196	Photosynthesis - antenna proteins - *Setaria italica*	Metabolism	Energy metabolism	1.49×10^{-4}	1.07×10^{-5}
	sita00360	Phenylalanine metabolism - *Setaria italica*	Metabolism	Amino acid metabolism	5.64×10^{-4}	4.57×10^{-5}
	sita00010	Glycolysis / Gluconeogenesis - *Setaria italica*	Metabolism	Carbohydrate metabolism	6.72×10^{-4}	6.05×10^{-5}
	sita00860	Porphyrin and chlorophyll metabolism - *Setaria italica*	Metabolism	Metabolism of cofactors and vitamins	8.04×10^{-4}	7.96×10^{-5}
	sita00970	Aminoacyl-tRNA biosynthesis - *Setaria italica*	Genetic Information Processing	Translation	1.08×10^{-3}	1.17×10^{-4}
	sita00030	Pentose phosphate pathway - *Setaria italica*	Metabolism	Carbohydrate metabolism	1.73×10^{-3}	2.03×10^{-4}
	sita00051	Fructose and mannose metabolism - *Setaria italica*	Metabolism	Carbohydrate metabolism	2.10×10^{-3}	2.65×10^{-4}
	sita00620	Pyruvate metabolism - *Setaria italica*	Metabolism	Carbohydrate metabolism	2.83×10^{-3}	3.88×10^{-4}
	sita00940	Phenylpropanoid biosynthesis - *Setaria italica*	Metabolism	Biosynthesis of other secondary metabolites	2.83×10^{-3}	4.07×10^{-4}
	sita00400	Phenylalanine, tyrosine and tryptophan biosynthesis - *Setaria italica*	Metabolism	Amino acid metabolism	3.29×10^{-3}	5.03×10^{-4}
	sita00220	Arginine biosynthesis - *Setaria italica*	Metabolism	Amino acid metabolism	4.36×10^{-3}	7.06×10^{-4}
	sita00260	Glycine, serine and threonine metabolism - *Setaria italica*	Metabolism	Amino acid metabolism	4.54×10^{-3}	7.77×10^{-4}
	sita00480	Glutathione metabolism - Setaria italica	Metabolism	Metabolism of other amino acids	1.29×10^{-2}	2.32×10^{-3}
	sita00520	Amino sugar and nucleotide sugar metabolism - *Setaria italica*	Metabolism	Carbohydrate metabolism	1.45×10^{-2}	2.74×10^{-3}
	sita00945	Stilbenoid, diarylheptanoid and gingerol biosynthesis - *Setaria italica*	Metabolism	Biosynthesis of other secondary metabolites	1.65×10^{-2}	3.27×10^{-3}
	sita00564	Glycerophospholipid metabolism - *Setaria italica*	Metabolism	Lipid metabolism	1.79×10^{-2}	3.72×10^{-3}
	sita00950	Isoquinoline alkaloid biosynthesis - *Setaria italica*	Metabolism	Biosynthesis of other secondary metabolites	3.29×10^{-2}	7.11×10^{-3}
	sita00053	Ascorbate and aldarate metabolism - *Setaria italica*	Metabolism	Carbohydrate metabolism	3.43×10^{-2}	7.72×10^{-3}

TS/TC	sita00360	Phenylalanine metabolism - *Setaria italica*	Metabolism	Amino acid metabolism	4.68×10^{-3}	4.46×10^{-5}
	sita04016	MAPK signaling pathway - plant - *Setaria italica*	Environmental Information Processing	Signal transduction	6.54×10^{-3}	1.50×10^{-4}
	sita00710	Carbon fixation in photosynthetic organisms - *Setaria italica*	Metabolism	Energy metabolism	6.54×10^{-3}	1.87×10^{-4}
	sita00330	Arginine and proline metabolism - *Setaria italica*	Metabolism	Amino acid metabolism	1.78×10^{-2}	6.79×10^{-4}
	sita00250	Alanine, aspartate and glutamate metabolism - *Setaria italica*	Metabolism	Amino acid metabolism	1.94×10^{-2}	9.25×10^{-4}
	sita00270	Cysteine and methionine metabolism - *Setaria italica*	Metabolism	Amino acid metabolism	4.52×10^{-2}	2.58×10^{-3}
	sita00950	Isoquinoline alkaloid biosynthesis - *Setaria italica*	Metabolism	Biosynthesis of other secondary metabolites	7.74×10^{-2}	5.32×10^{-3}
	sita00901	Indole alkaloid biosynthesis - *Setaria italica*	Metabolism	Biosynthesis of other secondary metabolites	7.74×10^{-2}	5.90×10^{-3}
	sita04075	Plant hormone signal transduction - *Setaria italica*	Environmental Information Processing	Signal transduction	9.02×10^{-2}	7.73×10^{-3}
	sita00965	Betalain biosynthesis - *Setaria italica*	Metabolism	Biosynthesis of other secondary metabolites	1.03×10^{-1}	9.79×10^{-3}
