# Supplementary material for: Transcriptome Analysis Reveals Molecular Mechanisms under Salt Stress in Leaves of Foxtail Millet (Setaria italica L.)
Source: Plants (Basel). 2022 Jul 18;11(14):1864. doi: 10.3390/plants11141864 (PMC9323065; doi:10.3390/plants11141864)
Supplement: Supplementary file 1 [file plants-11-01864-s001.zip › plants-1777431-Table S4.pdf]

Table S4. DGEs associated with photosynthesis under salt stress

Pathway	Function	Symbol	Name	Gene id	SS/SC		TS/TC	
					-Log10P	DEV	-Log10P	DEV
Photosynthesis - *Setaria italica* (foxtail millet)	Photosystem I	PsaD	photosystem I subunit II	101769456	7.60	-3.32		
Photosynthesis - *Setaria italica* (foxtail millet)	Photosystem I	PsaE	photosystem I subunit IV	101767783	3.05	-2.80		
Photosynthesis - *Setaria italica* (foxtail millet)	Photosystem I	PsaF	photosystem I subunit III	101783492	3.02	-3.12		
Photosynthesis - *Setaria italica* (foxtail millet)	Photosystem I	PsaG	photosystem I subunit V	101779542	6.55	-3.21		
Photosynthesis - *Setaria italica* (foxtail millet)	Photosystem I	PsaH	photosystem I subunit VI	101785215	3.69	-3.21		
Photosynthesis - *Setaria italica* (foxtail millet)	Photosystem I	PsaK	photosystem I subunit X	101755060	3.66	-3.11		
Photosynthesis - *Setaria italica* (foxtail millet)	Photosystem I	PsaL	photosystem I subunit XI	101786300	3.70	-2.95		
Photosynthesis - *Setaria italica* (foxtail millet)	Photosystem I	PsaN	photosystem I subunit PsaN	101759541	6.79	-2.79		
Photosynthesis - *Setaria italica* (foxtail millet)	Photosystem I	PsaO	photosystem I subunit PsaO	101771677	3.00	-4.26		
Photosynthesis - *Setaria italica* (foxtail millet)	Photosystem II	PsbO	photosystem II oxygen-evolving enhancer protein 1	101771243	3.00	-2.36		
Photosynthesis - *Setaria italica* (foxtail millet)	Photosystem II	PsbP	photosystem II oxygen-evolving enhancer protein 2	101777440	5.60	-2.68		
Photosynthesis - *Setaria italica* (foxtail millet)	Photosystem II	PsbP	photosystem II oxygen-evolving enhancer protein 2	101779739	4.01	-2.04		
Photosynthesis - *Setaria italica* (foxtail millet)	Photosystem II	PsbQ	photosystem II oxygen-evolving enhancer protein 3	101753687	4.93	-2.96		
Photosynthesis - *Setaria italica* (foxtail millet)	Photosystem II	PsbQ	photosystem II oxygen-evolving enhancer protein 3	101771660	3.97	-3.26		
Photosynthesis - *Setaria italica* (foxtail millet)	Photosystem II	PsbR	photosystem II 10kDa protein	101766967	4.71	-2.23		
Photosynthesis - *Setaria italica* (foxtail millet)	Photosystem II	PsbS	photosystem II 22kDa protein	101764210	2.71	-2.29		
Photosynthesis - *Setaria italica* (foxtail millet)	Photosystem II	PsbW	photosystem II PsbW protein	101778150	6.45	-2.70		

Photosynthesis - *Setaria italica* (foxtail millet)	Photosystem II	PsbW	photosystem II PsbW protein	101774285	3.38	-2.89		
Photosynthesis - *Setaria italica* (foxtail millet)	Photosystem II	PsbY	photosystem II PsbY protein	101775036	8.32	-3.17	2.04	-1.32
Photosynthesis - *Setaria italica* (foxtail millet)	Photosystem II	Psb27	photosystem II Psb27 protein	101763379	2.00	-2.53		
Photosynthesis - *Setaria italica* (foxtail millet)	Photosystem II	Psb28	photosystem II 13kDa protein	101784482	2.15	-2.91		
Photosynthesis - *Setaria italica* (foxtail millet)	F-type ATPase	AtpF	F-type H+-transporting ATPase subunit b	101770956	6.63	-2.75		
Photosynthesis - *Setaria italica* (foxtail millet)	F-type ATPase	AtpH	F-type H+-transporting ATPase subunit delta	101774771	4.54	-2.60		
Photosynthesis - *Setaria italica* (foxtail millet)	Photosynthethic electron transport	PetH	ferredoxin--NADP+ reductase [EC:1.18.1.2]	101763545	4.87	-2.28		
Photosynthesis - *Setaria italica* (foxtail millet)	Photosynthethic electron transport	PetH	ferredoxin--NADP+ reductase [EC:1.18.1.2]	101769250	3.75	-1.94		
Photosynthesis - *Setaria italica* (foxtail millet)	Photosynthethic electron transport	PetF	ferredoxin	101752738	8.34	-3.27		
Photosynthesis - *Setaria italica* (foxtail millet)	Photosynthethic electron transport	PetF	ferredoxin	101756065	2.21	-1.45		
Photosynthesis - *Setaria italica* (foxtail millet)	Photosynthethic electron transport	PetF	ferredoxin	101785087	3.98	-2.07		
Photosynthesis - *Setaria italica* (foxtail millet)	Photosynthethic electron transport	PetF	ferredoxin	101786886	2.12	-2.50		
Photosynthesis - *Setaria italica* (foxtail millet)	Photosynthethic electron transport	PetE	plastocyanin	101755940	3.29	-3.07		
Photosynthesis - *Setaria italica* (foxtail millet)	Cytochrome b6/f comples	PetC	cytochrome b6-f complex iron-sulfur subunit [EC:7.1.1.6]	101778821	5.67	-2.50		
Carbon fixation in photosynthetic organisms - *Setaria italica* (foxtail millet)	Calvin-Benson cycle	ALDO	fructose-bisphosphate aldolase, class I [EC:4.1.2.13]	101754815	5.32	-2.39		
Carbon fixation in photosynthetic organisms - *Setaria italica* (foxtail millet)	Calvin-Benson cycle	ALDO	fructose-bisphosphate aldolase, class I [EC:4.1.2.13]	101768485	4.47	-3.45	2.26	-1.47
Carbon fixation in photosynthetic organisms - *Setaria italica* (foxtail millet)	Calvin-Benson cycle	ALDO	fructose-bisphosphate aldolase, class I [EC:4.1.2.13]	101786189	4.50	-2.16	2.05	-1.26
Carbon fixation in photosynthetic organisms - *Setaria italica* (foxtail millet)	Calvin-Benson cycle	ALDO	fructose-bisphosphate aldolase, class I [EC:4.1.2.13]	101771498	2.09	1.36	2.07	1.37
Carbon fixation in photosynthetic organisms - *Setaria italica* (foxtail millet)	Calvin-Benson cycle	FBPase	fructose-1,6-bisphosphatase I [EC:3.1.3.11]	101755852	3.94	-2.01		

Carbon fixation in photosynthetic organisms - *Setaria italica* (foxtail millet)	Calvin-Benson cycle	FBPase	fructose-1,6-bisphosphatase I [EC:3.1.3.11]	101770545	6.94	-2.83		
Carbon fixation in photosynthetic organisms - *Setaria italica* (foxtail millet)	Calvin-Benson cycle	Transket lase	transketolase	101762694	2.81	-1.94		
Carbon fixation in photosynthetic organisms - *Setaria italica* (foxtail millet)	Calvin-Benson cycle	glpX-SEBP	fructose-1,6-bisphosphatase II / sedoheptulose-1,7-bisphosphatase [EC:3.1.3.11 3.1.3.37]	101753539	4.82	-2.32		
Carbon fixation in photosynthetic organisms - *Setaria italica* (foxtail millet)	Calvin-Benson cycle	RPI	ribose 5-phosphate isomerase A [EC:5.3.1.6]	101784778	4.90	-3.32		
Carbon fixation in photosynthetic organisms - *Setaria italica* (foxtail millet)	Calvin-Benson cycle	TPI	triosephosphate isomerase (TIM) [EC:5.3.1.1]	101773317	2.68	-1.95		
Carbon fixation in photosynthetic organisms - *Setaria italica* (foxtail millet)	Calvin-Benson cycle	TPI	triosephosphate isomerase (TIM) [EC:5.3.1.1]	101760718			3.58	-2.11
Carbon fixation in photosynthetic organisms - *Setaria italica* (foxtail millet)	Calvin-Benson cycle	GAPDH	glyceraldehyde-3-phosphate dehydrogenase (NADP+) (phosphorylating) [EC:1.2.1.13]	101752582	2.90	-2.82		
Carbon fixation in photosynthetic organisms - *Setaria italica* (foxtail millet)	Calvin-Benson cycle	GAPA	glyceraldehyde-3-phosphate dehydrogenase (NADP+) (phosphorylating) [EC:1.2.1.13]	101774537	2.63	-3.02		
Carbon fixation in photosynthetic organisms - *Setaria italica* (foxtail millet)	Calvin-Benson cycle	RPE	ribulose-phosphate 3-epimerase	101774062	5.31	-2.40		
Carbon fixation in photosynthetic organisms - *Setaria italica* (foxtail millet)	Calvin-Benson cycle	PRK	phosphoribulokinase [EC:2.7.1.19]	101762778	3.02	-1.72		
Carbon fixation in photosynthetic organisms - *Setaria italica* (foxtail millet)	Calvin-Benson cycle	PRK	phosphoribulokinase [EC:2.7.1.19]	101764636	4.53	-3.07		
Carbon fixation in photosynthetic organisms - *Setaria italica* (foxtail millet)	Calvin-Benson cycle	Rubisco	ribulose-bisphosphate carboxylase small chain [EC:4.1.1.39]	101763632	5.28	-2.38		
Carbon fixation in photosynthetic organisms - *Setaria italica* (foxtail millet)	Calvin-Benson cycle	Rubisco	ribulose-bisphosphate carboxylase small chain [EC:4.1.1.39]	101768770	2.10	-1.36		
Carbon fixation in photosynthetic organisms - *Setaria italica* (foxtail millet)	Calvin-Benson cycle	PGK	phosphoglycerate kinase [EC:2.7.2.3]	101765234	2.72	-2.86		
Carbon fixation in photosynthetic organisms - *Setaria italica* (foxtail millet)	C_4-Dicarboxylic acid cycle	PEPC	phosphoenolpyruvate carboxylase [EC:4.1.1.31]	101764206	4.92	-3.17		
Carbon fixation in photosynthetic organisms - *Setaria italica* (foxtail millet)	C_4-Dicarboxylic acid cycle	PPDK	pyruvate, orthophosphate dikinase [EC:2.7.9.1]	101760933	2.86	-1.64		

Carbon fixation in photosynthetic organisms - *Setaria italica* (foxtail millet)	C_4-Dicarboxylic acid cycle	MDH	malate dehydrogenase (oxaloacetate-decarboxylating)(NADP+) [EC:1.1.1.40]	101757986	2.57	9.55	4.22	11.82
Carbon fixation in photosynthetic organisms - *Setaria italica* (foxtail millet)	C_4-Dicarboxylic acid cycle	MDH	malate dehydrogenase (oxaloacetate-decarboxylating)(NADP+) [EC:1.1.1.40]	101777875	3.19	-1.75		
Carbon fixation in photosynthetic organisms - *Setaria italica* (foxtail millet)	C_4-Dicarboxylic acid cycle	MDH	malate dehydrogenase (oxaloacetate-decarboxylating)(NADP+) [EC:1.1.1.40]	101782922	3.63	4.50		
Carbon fixation in photosynthetic organisms - *Setaria italica* (foxtail millet)	C_4-Dicarboxylic acid cycle	MDH	malate dehydrogenase [EC:1.1.1.37]	101771167	2.54	-2.00		
Carbon fixation in photosynthetic organisms - *Setaria italica* (foxtail millet)	C_4-Dicarboxylic acid cycle	MDH	malate dehydrogenase [EC:1.1.1.37]	101771269	2.21	2.19		
Carbon fixation in photosynthetic organisms - *Setaria italica* (foxtail millet)	C_4-Dicarboxylic acid cycle	MDH2	malate dehydrogenase [EC:1.1.1.37]	101775144	4.48	-2.14		
Carbon fixation in photosynthetic organisms - *Setaria italica* (foxtail millet)	C_4-Dicarboxylic acid cycle	NADP-MDH	malate dehydrogenase (NADP+) [EC:1.1.1.82]	101775740	2.45	-2.59		
Carbon fixation in photosynthetic organisms - *Setaria italica* (foxtail millet)	C_4-Dicarboxylic acid cycle	NADP-MDH	malate dehydrogenase (NADP+) [EC:1.1.1.82]	101754520	2.72	-1.57		
Carbon fixation in photosynthetic organisms - *Setaria italica* (foxtail millet)	C_4-Dicarboxylic acid cycle	GGAT	glutamate--glyoxylate aminotransferase [EC:2.6.1.4 2.6.1.2 2.6.1.44]	101767913	2.46	-1.49		
