# Supplementary material for: Transcriptome Analysis Reveals Molecular Mechanisms under Salt Stress in Leaves of Foxtail Millet (Setaria italica L.)
Source: Plants (Basel). 2022 Jul 18;11(14):1864. doi: 10.3390/plants11141864 (PMC9323065; doi:10.3390/plants11141864)
Supplement: Supplementary file 1 [file plants-11-01864-s001.zip › plants-1777431-Table S5.pdf]

**Table S5.** DEGs related to transcription factor under salt stress

| Treatment | Gene id | Symbol | Family | $Log_{10}P$ | $log_2FoldChange$ | GO | KEGG |
|---|---|---|---|---|---|---|---|
| TS/TC | 101761227 | AIL5 | AP2/ERF | $7.99×10^{−3}$ | Inf | GO:0007275; GO:0006355; GO:0003677; GO:0003700; GO:0005634 | K09285 |
| | 101773645 | ERF105 | AP2/ERF | $1.28×10^{−5}$ | 2.48 | GO:0006351 | K09286 |
| | 101777485 | ERF2 | AP2/ERF | $9.60×10^{−4}$ | 2.56 | GO:0006355; GO:0003700; GO:0003677; GO:0005634 | K09286 |
| | 101784800 | ERF094 | AP2/ERF | $5.12×10^{−4}$ | 4.05 | GO:0006351 | |
| | 101785798 | bHLH48 | bHLH | $5.46×10^{−3}$ | -2.36 | GO:0046983; GO:0003677 | |
| | 101755679 | UNE10 | bHLH | $9.41×10^{−4}$ | 1.76 | GO:0009506; GO:0046983; GO:0009567 | |
| | 101765898 | bHLH112 | bHLH | $5.95×10^{−3}$ | 1.54 | GO:0001228; GO:0045944; GO:0001046; GO:0046983; GO:0005634 | |
| | 101780831 | bHLH041 | bHLH | $1.14×10^{−3}$ | 2.21 | GO:0046983 | |
| | 101753232 | HHO5 | GARP | $5.51×10^{−3}$ | -2.19 | GO:0006355; GO:0003677; GO:0005634 | |
| | 101759317 | Hsf C-2b | HSF | $3.35×10^{−4}$ | -2.00 | GO:0006355; GO:0043565; GO:0003700; GO:0005634 | K09419 |
| | 101770723 | Hsf C-2a | HSF | $4.05×10^{−4}$ | -2.94 | GO:0003677 | K09419 |
| | 101768861 | PHL11 | MYB-MYC | $8.11×10^{−3}$ | -2.55 | GO:0006355; GO:0003677; GO:0005634 | |
| | 101773024 | MYC2 | MYB-MYC | $1.38×10^{−4}$ | -7.12 | GO:0046983 | |
| | 101774132 | NAC104 | NAC | $3.03×10^{−4}$ | -3.42 | GO:0048367; GO:0006355; GO:0003677; GO:0010089; GO:0043067; GO:0005634 | |
| | 101777848 | NAC72 | NAC | $2.64×10^{−3}$ | -2.44 | GO:0006355; GO:0003677; GO:0005634 | |
| | 101786033 | JUB 1 | NAC | $6.38×10^{−3}$ | -3.04 | GO:0006355; GO:0003677; GO:0005634 | |
| | 101785579 | NF-Y | NF | $6.06×10^{−4}$ | -Inf | GO:0005634; GO:0046982 | K08065 |
| | 101767348 | PCL1 | PCL | $7.29×10^{−3}$ | -1.98 | GO:0006355; GO:0003677; GO:0005634 | |
| | 101763706 | GTL1 | Trihelix | $5.59×10^{−3}$ | -1.91 | GO:0003677 | |
| | 101756467 | WRKY33 | WRKY | $6.48×10^{−3}$ | 1.66 | GO:0006355; GO:0043565; GO:0003700 | |
| | 101756513 | WRKY57 | WRKY | $9.73×10^{−4}$ | 4.62 | GO:0006355; GO:0043565; GO:0003700 | |
| | 101758356 | WRKY70 | WRKY | $6.44×10^{−3}$ | 1.43 | GO:0006355; GO:0043565; GO:0003700 | |
| | 101762911 | WRKY70 | WRKY | $5.11×10^{−3}$ | 1.64 | GO:0006355; GO:0043565; GO:0003700 | |
| | 101769236 | WRKY4 | WRKY | $6.75×10^{−3}$ | 1.84 | GO:0006355; GO:0043565; GO:0003700 | |

| | | | | | | | |
|---|---|---|---|---|---|---|---|
| | 101769849 | WRKY50 | WRKY | $8.00\times10^{-3}$ | 2.09 | GO:0006355; GO:0043565; GO:0003700 | |
| | 101775299 | WRKY53 | WRKY | $8.74\times10^{-3}$ | 1.85 | GO:0006355; GO:0043565; GO:0003700 | |
| | 101776857 | WRKY26 | WRKY | $1.33\times10^{-3}$ | 1.82 | GO:0006355; GO:0043565; GO:0003700 | K13424 |
| | 101781477 | WRKY28 | WRKY | $6.80\times10^{-4}$ | 3.35 | GO:0006355; GO:0043565; GO:0003700 | |
| | 101782717 | WRKY24 | WRKY | $3.27\times10^{-3}$ | 1.74 | GO:0006355; GO:0043565; GO:0003700 | K13424 |
| | 101771359 | ERF018 | AP2/ERF | $9.81\times10^{-3}$ | -Inf | GO:0006355; GO:0003700; GO:0003677; GO:0005634 | |
| | 101776627 | ERF034 | AP2/ERF | $3.48\times10^{-3}$ | -4.7 | GO:0006355; GO:0003700; GO:0003677; GO:0005634; GO:0009536 | K09286 |
| | 101777340 | ERF018 | AP2/ERF | $2.88\times10^{-3}$ | -4.77 | GO:0006355; GO:0003700; GO:0003677; GO:0005634 | |
| | 101778978 | ERF105 | AP2/ERF | $9.67\times10^{-3}$ | -1.72 | GO:0006355; GO:0003700; GO:0003677; GO:0005634 | K09286 |
| | 101765739 | bHLH35 | bHLH | $2.93\times10^{-3}$ | -2.44 | GO:0046983 | |
| | 101766425 | bHLH35 | bHLH | $1.91\times10^{-7}$ | -3.1 | GO:0046983 | |
| | 101766960 | bHLH35 | bHLH | $6.48\times10^{-6}$ | -2.83 | GO:0046983 | |
| | 101773457 | APG | bHLH | $4.13\times10^{-3}$ | -1.87 | GO:0031539; GO:0042802; GO:0003677; GO:0046983; GO:0009740; GO:0010017; GO:0005634; GO:0009704 | K12126 |
| | 101781503 | bHLH30 | bHLH | $1.64\times10^{-3}$ | -2.17 | GO:0046983; GO:0005634; GO:0003677 | |
| | 101757337 | bHLH128 | bHLH | $3.90\times10^{-4}$ | 1.95 | GO:0001228; GO:0001046; GO:0046983; GO:0005634; GO:0006366 | |
| SS/SC | 101760887 | BHLH089 | bHLH | $2.12\times10^{-5}$ | 2.27 | GO:0046983 | |
| | 101761750 | BHLH062 | bHLH | $8.86\times10^{-3}$ | 1.4 | GO:0046983 | |
| | 101764187 | ILI3 | bHLH | $9.51\times10^{-3}$ | 3.91 | GO:0046983 | |
| | 101767345 | bHLH112 | bHLH | $8.06\times10^{-3}$ | 1.42 | GO:0046983 | |
| | 101768277 | bHLH68 | bHLH | $5.24\times10^{-3}$ | 1.54 | GO:0046983 | |
| | 101775649 | PIF4 | bHLH | $6.68\times10^{-3}$ | 1.42 | GO:0046983 | |
| | 101755679 | UNE10 | bHLH | $6.21\times10^{-7}$ | 2.65 | GO:0009506; GO:0046983; GO:0009567 | |
| | 101756519 | HBP-1a | bZIP | $8.04\times10^{-3}$ | 1.67 | GO:0006355; GO:0043565; GO:0003700 | K09060 |
| | 101760850 | HY5 | bZIP | $2.24\times10^{-4}$ | 2.02 | GO:0006355; GO:0043565; GO:0003700 | K16241 |
| | 101785019 | RL9 | GARP | $2.08\times10^{-3}$ | 1.69 | GO:0009944; GO:0048481; GO:0044212; GO:0009887; GO:0006355; GO:0010051; GO:0010158; GO:0009956; GO:0005634 | |

| | | | | | | |
|---|---|---|---|---|---|---|
| 101752777 | GATA23 | GATA | $4.72\times10^{-4}$ | 1.87 | GO:0006366 | |
| 101753493 | GATA18 | GATA | $5.55\times10^{-3}$ | 1.45 | GO:0001085; GO:0008270; GO:0001228; GO:0045944; GO:0005667; GO:0000977; GO:0030154; GO:0003682; GO:0005634 | |
| 101759317 | HSF C-2b | HSF | $2.10\times10^{-7}$ | -2.79 | GO:0006355; GO:0043565; GO:0003700; GO:0005634 | K09419 |
| 101767343 | HSF C-1b | HSF | $2.49\times10^{-5}$ | -2.60 | GO:0006355; GO:0043565; GO:0006970; GO:0003700; GO:0009737; GO:0005634 | K09419 |
| 101769419 | HSF B-2a-like | HSF | $3.22\times10^{-3}$ | -2.32 | GO:0006355; GO:0043565; GO:0003700; GO:0005634 | K09419 |
| 101770723 | HSF C-2a-like | HSF | $1.21\times10^{-9}$ | -3.82 | GO:0003677 | K09419 |
| 101774980 | HSFA-2c | HSF | $8.40\times10^{-4}$ | -1.84 | GO:0042802; GO:0006355; GO:0043565; GO:0003700; GO:0005634 | |
| 101763570 | MADS-Box51 | MADS-Box | $1.67\times10^{-3}$ | 1.72 | GO:0006355; GO:0003677; GO:0046983; GO:0005634 | |
| 101773024 | MYC2 | MYB-MYC | $9.67\times10^{-3}$ | -5.11 | GO:0046983 | |
| 101773784 | APL | MYB-MYC | $5.54\times10^{-3}$ | -1.95 | GO:0006355; GO:0003677; GO:0005634 | |
| 101770921 | RAX2 | MYB-MYC | $4.54\times10^{-3}$ | -1.60 | GO:0003677 | K09422 |
| 101755975 | DIVARICATA | MYB-MYC | $4.83\times10^{-3}$ | 1.50 | GO:0006355; GO:0003677; GO:0005634 | |
| 101770038 | MYBS3 | MYB-MYC | $5.87\times10^{-3}$ | 1.76 | GO:0008270; GO:0004402; GO:0006357; GO:0003713; GO:0003677; GO:0006338; GO:0003682; GO:0035066; GO:0005634; | |
| 101771187 | SRM1 | MYB-MYC | $1.44\times10^{-5}$ | 2.54 | GO:0006355; GO:0003677; GO:0005634 | |
| 101774132 | NAC104 | NAC | $5.61\times10^{-4}$ | -3.98 | GO:0048367; GO:0006355; GO:0003677; GO:0010089; GO:0043067; GO:0005634 | |
| 101767581 | NF-Y subunit A-4 | NF | $2.19\times10^{-3}$ | -1.77 | GO:0006355; GO:0003677; GO:0016602; GO:0003700 | K08064 |
| 101770060 | NF-Y subunit B-11 | NF | $5.06\times10^{-3}$ | 1.64 | GO:0006355; GO:0043565; GO:0005634; GO:0046982 | K08065 |
| 101760005 | NF-Y subunit C-2 | NF | $6.33\times10^{-5}$ | 2.58 | GO:0005634; GO:0046982 | |
| 101762971 | NF-Y subunit B-8 | NF | $5.97\times10^{-3}$ | 2.81 | GO:0006355; GO:0043565; GO:0005634; GO:0046982 | K08065 |
| 101771780 | PCF7 | TCP | $1.19\times10^{-3}$ | -2.15 | GO:0016020 | |
| 101771904 | TGAL1 | TGA | $1.65\times10^{-3}$ | -2.77 | GO:0018105; GO:0046777; GO:0035556; GO:0005516; GO:0005509; GO:0009931; GO:0006355; GO:0009738; GO:0043565; GO:0005524; GO:0005737; GO:0003700; GO:0004683; GO:0005634 | K14431 |
| 101756052 | WRKY63 | WRKY | $3.05\times10^{-3}$ | -Inf | GO:0006355; GO:0043565; GO:0003700 | |
| 101757145 | WRKY57 | WRKY | $1.75\times10^{-3}$ | -1.93 | GO:0006355; GO:0043565; GO:0003700 | |
| 101765520 | WRKY14 | WRKY | $7.86\times10^{-3}$ | -3.58 | GO:0006355; GO:0043565; GO:0003700 | |

| | | | | | |
|---|---|---|---|---|---|
| 101769639 | WRKY38 | WRKY | $1.43\times10^{-3}$ | -4.20 | GO:0006355; GO:0043565; GO:0003700 |
| 101772113 | WRKY70 | WRKY | $5.75\times10^{-3}$ | -Inf | GO:0006355; GO:0043565; GO:0003700 |
| 101774144 | WRKY58 | WRKY | $3.38\times10^{-3}$ | -5.48 | GO:0006355; GO:0043565; GO:0003700 |
| 101756467 | WRKY33 | WRKY | $8.46\times10^{-4}$ | 1.83 | GO:0006355; GO:0043565; GO:0003700 |
