# Supplementary material for: Transcriptome Analysis Reveals Molecular Mechanisms under Salt Stress in Leaves of Foxtail Millet (Setaria italica L.)
Source: Plants (Basel). 2022 Jul 18;11(14):1864. doi: 10.3390/plants11141864 (PMC9323065; doi:10.3390/plants11141864)
Supplement: Supplementary file 1 [file plants-11-01864-s001.zip › plants-1777431-Table S6.pdf]

Table S6. Sequencing statistics of RNA libraries.

Sample	Reads No.	Bases (Gp)	Q20 (%)	Q30 (%)	Clean Reads No.	Clean Data (Gp)	Clean Reads (%)	Clean Data (%)	Total Mapped rate (%)	Uniquely Mapped rate (%)
SS1	43275738	6.53	97.64	94.15	43051268	6.48	99.48	99.19	91.79	95.14
SS2	44032306	6.65	97.39	93.54	43798096	6.60	99.46	99.20	90.17	95.96
SS3	46559864	7.03	97.63	94.12	46311302	6.97	99.46	99.18	92.19	94.42
TS1	52169882	7.88	97.62	94.14	51892368	7.81	99.46	99.18	91.81	95.46
TS2	40160758	6.06	97.63	94.12	39956892	6.02	99.49	99.19	91.14	95.31
TS3	45878970	6.93	97.62	94.08	45648738	6.87	99.49	99.22	93.27	93.88
SC1	50118866	7.57	97.72	94.29	49886262	7.51	99.53	99.26	92.76	96.06
SC2	40694258	6.14	97.73	94.33	40503578	6.10	99.53	99.27	93.07	96.42
SC3	42262684	6.38	97.58	94.01	42038368	6.33	99.46	99.22	92.46	96.03
TC1	43059668	6.50	97.67	94.20	42845472	6.45	99.50	99.24	91.92	95.41
TC2	45550060	6.88	97.71	94.26	45333066	6.83	99.52	99.24	92.66	95.27
TC3	41427198	6.26	97.77	94.43	41258604	6.21	99.59	99.33	91.32	95.50
Total	535190252	80.81	-	-	532524014	80.19	-	-	91.79	95.14

Note: Q20 (%): percentage of bases whose base recognition accuracy is more than 99%; Q30 (%): percentage of bases whose base recognition accuracy is more than 99.9%.
