# Supplementary material for: Transcriptome Analysis Reveals Molecular Mechanisms under Salt Stress in Leaves of Foxtail Millet (Setaria italica L.)
Source: Plants (Basel). 2022 Jul 18;11(14):1864. doi: 10.3390/plants11141864 (PMC9323065; doi:10.3390/plants11141864)
Supplement: Supplementary file 1 [file plants-11-01864-s001.zip › plants-1777431-Table S7.pdf]

Table S7. 104 foxtail millet accessions for testing in the study

Code	Name	Origin	Status
FM1	Dong gu No.1	Shenyang, Liaoning Province	Commodity seeds
FM2	Jin gu No.2	Siping, Jilin Province	Commodity seeds
FM3	Dong gu No.3	Shenyang, Liaoning province	Commodity seeds
FM4	Jin gu 21	Jinzhong, Shanxi Province	Commodity seeds
FM5	Jing dou dun gu No.8	Beijing	Commodity seeds
FM6	Hong gu 2000	Fuxin, Liaoning Province	Commodity seeds
FM7	Qin zhou huang gu zi	Jinzhong, Shanxi Province	Commodity seeds
FM8	Huang jin gu zi	Shenyang, Liaoning province	Commodity seeds
FM9	Dun gu	Zhangye, Gansu Province	Commodity seeds
FM10	Niao bu dan	Zhangye, Gansu Province	Commodity seeds
FM11	Lü bao gan jin gu	Jinzhong, Shanxi Province	Commodity seeds
FM12	Huang jin wang gu	Chaoyang, Liaoning Province	Self-reservation seeds
FM13	Feng hong gu	Chifeng, Inner Mongolia Province	Commodity seeds
FM14	Chi gu No.16	Chifeng, Inner Mongolia Province	Commodity seeds
FM15	Chi gu No.10	Chifeng, Inner Mongolia Province	Commodity seeds
FM16	Liao gu No.4	Shenyang, Liaoning province	Commodity seeds
FM17	GE07 fu xin	Fuxin, Liaoning Province	Self-reservation seeds
FM18	Liao gu No.2	Shenyang, Liaoning province	Commodity seeds
FM19	Liao feng gu No.3	Dandong, Liaoning Province	Commodity seeds
FM20	Zhang gu 1501	Shenyang, Liaoning province	Self-reservation seeds
FM21	Ge 1505	Fuxin, Liaoning Province	Self-reservation seeds
FM22	Jing xuan shu xiang zao gan gu	Handan, Hebei Province	Commodity seeds
FM23	Hong dun gu	Beijing	Commodity seeds
FM24	Jin wa wa	Zhengzhou, Henan Province	Commodity seeds
FM25	Ji gu No.19	Handan, Hebei Province	Commodity seeds
FM26	Ji gu No.1860	Handan, Hebei Province	Commodity seeds

FM27	Niao bu diao zhan xiang gu	Hebi City, Henan Province	Commodity seeds
FM28	Shu xiang No.1	Handan, Hebei Province	Commodity seeds
FM29	Shu xiang No.2	Handan, Hebei Province	Commodity seeds
FM30	Xiang zhan niao bu zhuo	Beijing	Commodity seeds
FM31	Te gong hong xiang gu	Handan, Hebei Province	Commodity seeds
FM32	Te han No.1	Handan, Hebei Province	Commodity seeds
FM33	Niao bu chi hong gai gu	Xinyang, Henan Province	Commodity seeds
FM34	Dong chang No.1	Handan, Hebei Province	Commodity seeds
FM35	Dun xiang No.3	Jinan, Shandong Province	Commodity seeds
FM36	Jin gu wang	Beijing	Commodity seeds
FM37	Huang jin dun gu	Botou, Hebei Province	Commodity seeds
FM38	Dun gu No.1	Fuyu, Jilin Province	Commodity seeds
FM39	Nei meng gu	Chifeng, Inner Mongolia Province	Self-reservation seeds
FM40	Jin gu No.8	Baoding, Hebei Province	Commodity seeds
FM41	Bo cai bi	Dongying, Shandong Province	Self-reservation seeds
FM42	Hong gu	Shenyang, Liaoning province	Self-reservation seeds
FM43	Qin zhou huang	Shenyang, Liaoning province	Commodity seeds
FM44	Chai jian	Jinan, Shandong Province	Self-reservation seeds
FM45	Ji gu No.19	Jinan, Shandong Province	Commodity seeds
FM46	Ji gu No.20	Jinan, Shandong Province	Commodity seeds
FM47	Ji you jin gu fu er	Beijing	Commodity seeds
FM48	Jing xuan dun gu yi hao	Cangzhou, Hebei Province	Commodity seeds
FM49	Zhang lan jun1 liu zhong	Dongying, Shandong Province	Self-reservation seeds
FM50	Yu shi qin liu zhong	Dongying, Shandong Province	Self-reservation seeds
FM51	Jin gu No.54	Luliang, Shanxi Province	Commodity seeds
FM52	Jin fen No.106	Yan'an, Shanxi Province	Commodity seeds
FM53	Jin gu No.57	Luliang, Shanxi Province	Commodity seeds
FM54	Jin gu No.40	Luliang, Shanxi Province	Commodity seeds

FM55	Fen xuan No.3	Luliang, Shanxi Province	Commodity seeds
FM56	Jin zhen zhu	Luliang, Shanxi Province	Commodity seeds
FM57	Jin gu No.29	Luliang, Shanxi Province	Commodity seeds
FM58	Jin fen No.2	Yan'an, Shanxi Province	Commodity seeds
FM59	Fen te No.11	Luliang, Shanxi Province	Commodity seeds
FM60	T6	Lianyungang, Jiangsu Province	Self-reservation seeds
FM61	T7	Linyi, Shandong Province	Self-reservation seeds
FM62	T3	Linyi, Shandong Province	Self-reservation seeds
FM63	T2	Linyi, Shandong Province	Self-reservation seeds
FM64	Hong xiang zhuang yuan	Linyi, Shandong Province	Self-reservation seeds
FM65	T5	Linyi, Shandong Province	Self-reservation seeds
FM66	T9	Linyi, Shandong Province	Self-reservation seeds
FM67	T10	Linyi, Shandong Province	Self-reservation seeds
FM68	Huang jin gong gu	Linyi, Shandong Province	Self-reservation seeds
FM69	T8	Linyi, Shandong Province	Self-reservation seeds
FM70	Ma zhuang liu	Tai'an, Shandong Province	Commodity seeds
FM71	Ji gu No.16	Jining, Shandong Province	Self-reservation seeds
FM72	Xiao zao gu	Beijing	Commodity seeds
FM73	Liu yi gu	Beijing	Commodity seeds
FM74	Bai ba zi	Beijing	Commodity seeds
FM75	Cha dian gu	Tianjin	Commodity seeds
FM76	Mao mao gu	Tianjin	Commodity seeds
FM77	Dan shen han gu	Tianjin	Commodity seeds
FM78	Da mao gu	Hebei Baoji	Commodity seeds
FM79	Qian chuan jin gu	Langfang, Hebei Province	Commodity seeds
FM80	Sheng tou jin	Langfang, Hebei Province	Commodity seeds
FM81	Qi tou bai yang wei ba gu	Langfang, Hebei Province	Commodity seeds
FM82	Qi tou ding gu zi	Binzhou, Shandong Province	Commodity seeds

FM83	Jin de	America	Commodity seeds
FM84	Bai fan gu	Xianyang, Shaanxi Province	Commodity seeds
FM85	Dao ba qi	Beijing	Commodity seeds
FM86	Xiao huang gu	Beijing	Commodity seeds
FM87	Ga ga qing	Beijing	Commodity seeds
FM88	Bai gu san	Beijing	Commodity seeds
FM89	Hong gen gu zi	Beijing	Commodity seeds
FM90	Pu huang yu	Beijing	Commodity seeds
FM91	Zhu wei ba xi	Beijing	Commodity seeds
FM92	Wan ping bai gu	Beijing	Commodity seeds
FM93	Tong zhou zhong	Beijing	Commodity seeds
FM94	San bian se	Beijing	Commodity seeds
FM95	Yan xia gu 1hao	Yan'an, Shaanxi Province	Commodity seeds
FM96	Zhu xi No.13	Zhangjiakou, Hebei Province	Commodity seeds
FM97	Jin dian huang	Zhangjiakou, Hebei Province	Commodity seeds
FM98	Xiao huang gu	Daxing'anling, Heilongjiang Province	Commodity seeds
FM99	Er huang gu	Hohhot, Inner Mongolia Province	Commodity seeds
FM100	Zhang nong No.1	Zhangjiakou, Hebei Province	Commodity seeds
FM101	Cheng nong No.2	Zhangjiakou, Hebei Province	Commodity seeds
FM102	Huang miao da hua pi	Beijing	Commodity seeds
FM103	Mo li gu	Beijing	Commodity seeds
FM104	Lan gu No.3	Zibo, Shandong Province	Commodity seeds
